# Harnessing Advances in Bone Tissue Engineering for Design of Bone‐on‐Chip Systems

**DOI:** 10.1002/adhm.202503525

**Published:** 2025-11-17

**Authors:** Farhad Sanaei, Yannick Hajee, Gerry L. Koons, David T. Wu, Sander C.G. Leeuwenburgh, Jeroen J.J.P. van den Beucken, Mani Diba

**Affiliations:** ^1^ Regenerative Biomaterials Dentistry Radboud Institute for Medical Innovation Radboud University Medical Center Nijmegen 6525 EX The Netherlands; ^2^ Computational Pathology Group Department of Pathology Radboud University Medical Center Nijmegen 6525 GA The Netherlands; ^3^ Harvard John A. Paulson School of Engineering and Applied Sciences Harvard University Boston 02138 USA; ^4^ Wyss Institute for Biologically Inspired Engineering Harvard University Boston 02215 USA; ^5^ Department of Oral Medicine Infection, and Immunity Harvard School of Dental Medicine Boston 02115 USA; ^6^ Faculty of Dental Medicine and Oral Health Sciences McGill University Montreal H3A1G1 Canada

**Keywords:** BoC design, BoCs, bone, bone tissue engineering, bone‐on‐chips, organ‐on‐chip, perspective

## Abstract

Organ‐on‐Chip systems demonstrate significant potential as next‐generation models to study human (patho)physiology and to assess new therapies. Whereas on‐chip models of soft tissues and organs have progressed substantially over the past decade, the development of bone‐on‐chip (BoC) systems remains comparatively slower. This slower progress stems from the structural and functional complexity of bone tissue, which hampers efforts to recapitulate bone (patho)physiology on‐chip. Advances in bone tissue engineering (BTE) now provide opportunities regarding i) innovative biomaterials design strategies and ii) advanced bioengineering tools, enabling a closer replication of the architectural and functional complexity of native bone. However, these technological advances have primarily resulted in improved regenerative therapies, while also offering opportunities for innovative BoC designs. This perspective article identifies key requirements for BoCs, explores existing models and their respective advantages and limitations. Subsequently, opportunities derived from BTE are highlighted to accelerate the development of BoCs which more accurately capture crucial features of in vivo bone (patho)physiology. Finally, key considerations for BoC design are discussed, and an outlook for further developments in this emerging field is provided.

## Graphical abstract

Bone‐on‐chip (BoC) systems demonstrate significant potential as next‐generation models to study human (patho)physiology and evaluate new therapies. However, progress toward functional, human‐like BoCs has been hindered by the structural and functional complexity of bone. This perspective discusses how insights from bone tissue engineering can guide BoC designs that better capture in vivo bone (patho)physiology, accelerating therapeutic development and disease modeling.

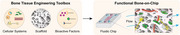

## 1 Introduction

An increasing body of evidence shows that correlation between traditional in vitro and in vivo animal models and human (patho)physiology is poor.^[^ ^1^ ^]^ In vitro studies do not capture the complexity and dynamicity of human tissues and organs, while in vivo studies raise ethical concerns and are hampered by interspecies differences. These translational challenges currently hinder clinical translatability of results obtained from these models and limit their scientific applicability and societal impact, emphasizing the need for alternatives. Organ‐on‐chip (OoC) systems are emerging as advanced alternatives to these models.^[^ ^2^ ^]^ Remarkably, as evidenced by the 2022 U.S. Food and Drug Administration (FDA) Modernization Act 2.0,^[^ ^3^ ^]^ (inter)national regulatory agencies have recently begun to allow for in vitro alternatives for animal testing of new therapies, which opens new avenues for the scientific and clinical impact of OoCs.

Although substantial progress has been made for on‐chip versions of several types of tissues and organs such as liver and lung,^[^ ^2^ ^]^ bone‐on‐chip (BoC) systems remain largely underdeveloped. This stunted progress is mainly caused by the structural and functional complexities of bone. These complexities include bone's hierarchical structure, mineralized extracellular matrix (ECM), heterogenous cell population, unique mechanical properties, and complex responses to dynamic loading.

Over the past three decades, the field of bone tissue engineering (BTE) has witnessed significant progress through advances in bioengineering complex aspects of bone tissue.^[^ ^4^ ^]^ However, these advances have largely been focused on bone regeneration.^[^ ^4^ ^]^ We foresee currently available BTE tools can substantially benefit the development of BoCs. Such advances in BoC will enhance our ability to decipher key aspects of specific (patho)physiological processes in bone formation, bone resorption, and bone remodeling, thereby contributing to both fundamental research and therapeutic development.

However, progress in BoCs first requires understanding the specific bone features that are the targets needed for near‐physiological BoCs. This understanding can guide identification of tools and solutions from the BTE and OoC fields to advance BoCs. To achieve this objective, it is essential to clearly define what constitutes a BoC system and delineate the scope of relevant models. In this perspective, we focus specifically on BoC platforms defined as OoC systems incorporating osteoblasts, osteoclasts, and/or osteocytes to emulate key bone‐specific functions in vitro. More broadly, we define OoC systems as microengineered in vitro models featuring fluidic components and designed to replicate specific organ‐level physiology.

This perspective article differs from prior review articles, such as that of Owen and Reilly (2018)^[^ ^5^ ^]^ which focus on a broader range of in vitro models of bone remodeling and emphasize osteoblast–osteoclast co‐culture as a minimal requirement. Additionally, bone marrow‐on‐chip systems, which serve distinct biological purposes, are considered out of scope for this discussion and are reviewed comprehensively elsewhere.^[^ ^6^ ^]^ By narrowing our focus, we aim to identify how targeted advances in BTE—particularly in biomaterials and biofabrication—can address the current limitations of BoCs and inform rational design of next‐generation systems.

In the following sections, this perspective progresses from discussing the fundamental building blocks of bone (Section 2) to tissue‐level systems (Section 3). These sections frame the classical BTE triad of scaffolds, bioactive cues, and progenitor cells (Sections 2.1–2.3), but also delve into newer BTE developments such as the importance of dynamic mechanical environment (Section 2.4) and multi‐tissue systems (Sections 3.2–3.3).

## 2 Foundational Features of Native Bone

**Figure 1:**
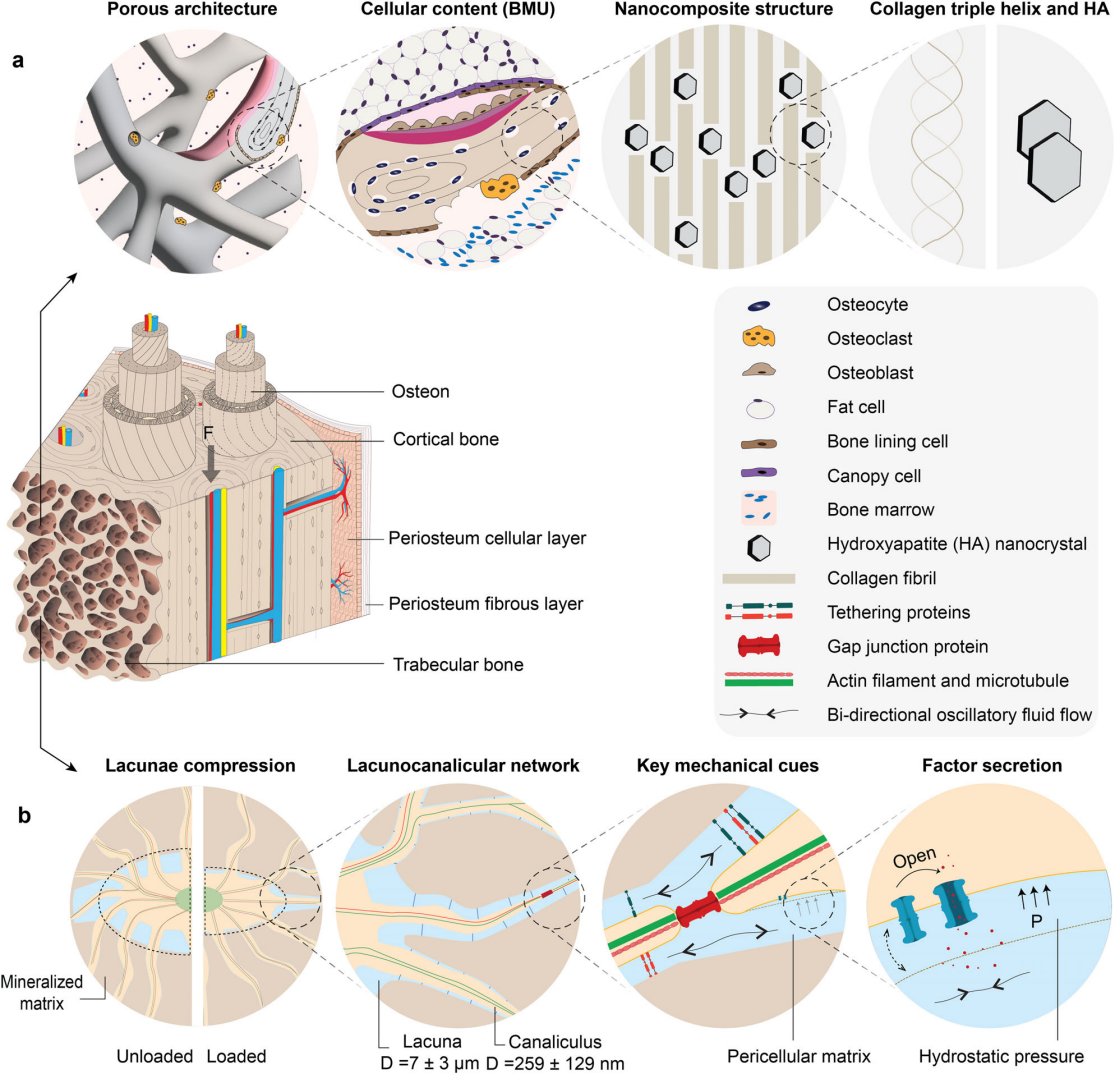
Overview of key physiological features of bone for recapitulation in BoCs. Long bones are composed of distinct concentric layers spanning radially from the periosteum to trabeculae. 1) The periosteum, a thin layer of connective tissue, envelops the outer surface of all bones except at joints. 2) The cortical bone, characterized by its high density and degree of organization, and 3) the trabecular bone, characterized by its spongy architecture which houses bone marrow. Both bone types, cortical and trabecular, are composed of osteons, which are cylindrical units of bone tissue. a) Bone architecture from macro‐ to nanoscale. Trabecular bone has macropores (0.1–1 mm) which accommodate bone marrow that supports hematopoiesis. Osteons (100–400 µm) undergo continual remodeling through the orchestrated actions of three main bone cell types within the basic multicellular unit (BMU). The BMU is a temporary, spatially organized structure that coordinates the sequential resorption and formation of bone, thereby maintaining bone health and function. The three main cell types in the BMU are osteoblasts, osteoclasts, and osteocytes, which are mainly responsible for bone formation, resorption, and maintenance, respectively. Other supporting cell populations also play key roles in bone health and remodeling. Bone lining cells, flattened osteoblast‐derived cells covering quiescent bone surfaces, can revert to active osteoblasts and contribute to coupling resorption with formation. Canopy cells, derived from osteoblast progenitors, colonize resorbed bone surfaces, protecting the site during new bone deposition.^[^ ^27^ ^]^Osteons follow a lamellar pattern during formation, wherein collagen fibers (1–10 µm) are deposited in alternating orientations. This structural pattern significantly fortifies lamellar bone. At the nanoscale, bone is composed of collagen fibrils and hydroxyapatite (HA) nanocrystals, forming a nanocomposite which contributes to its unique mechanical properties.^[^ ^12^ ^]^ b) Translation of external load to internal mechanotransduction of bone through key pathways. Osteocytes – derived from osteoblasts trapped in a mineralized matrix– reside within small cavities called lacunae, situated between lamellae, and are connected through a network of tunnels known as canaliculi, collectively forming the lacunocanalicular network (LCN). Native bone experiences cyclic mechanical loading as a result of human locomotion, inducing pulsatile fluid flow in the LCN through pericellular matrix pores. This flow can be sensed by the osteocytes through various sensing mechanisms such as actin stretch,^[^ ^28^ ^]^ mechanosensitive ion channels,^[^ ^29^ ^]^ and fluid drag force on the tethering proteins.^[^ ^28^ ^]^

Understanding the foundational features of healthy bone is key to designing near‐physiological BoCs. Bone is a complex, hierarchical tissue composed of an organic‐inorganic nanocomposite matrix that hosts a variety of cell types. Physiological features of bone are also dynamic, and its unique properties and functions are significantly influenced by mechanical cues and its multi‐scale hierarchy (**Figure** **1**). While these features are often essential for mimicking human bone tissue, their dynamic nature presents significant challenges for recapitulation in BoCs.

### 2.1 Architecture

#### 2.1.1 Architectural Organization of Native Bone

Designing BoCs that faithfully mimic bone physiology requires recapitulating key architectural features of bone, such as its 3D structure, hierarchical organization, porosity, and anisotropy (Figure 1). 2D microfluidic systems typically rely on monolayer cultures of cells within microfluidic channels,^[^ ^7^, ^8^, ^9^ ^]^ and therefore do not accurately replicate the 3D environment of bone. However, a recent paper reports the 2D‐to‐3D transition of a BoC based on osteoblastic differentiation of mesenchymal stromal cell (MSC) monolayers followed by their 3D self‐assembly into a tissue‐like mineralized structure.^[^ ^10^ ^]^ These 3D structures are reminiscent of organoids and suggest that cellular self‐assembly may be a viable strategy to achieve certain levels of 3D complexity. Other cellular assemblies include an organoid system resembling woven bone, which lacks mature bone's 3D structure regarding collagen organization.^[^ ^11^ ^]^ Overall, we postulate that BoCs employing 3D ECM mimics potentially offer a higher degree of structural control than systems solely relying on cellular self‐assembly.

#### 2.1.2 Porosity and Surface Topology

Furthermore, porosity is a particularly important feature for trabecular bone (Figure 1a), which for instance houses bone marrow and generates a high surface‐to‐volume ratio for rapid remodeling and calcium homeostasis.^[^ ^12^ ^]^ To target this feature, current BoCs employ porous scaffolds made from synthetic polymers,^[^ ^13^, ^14^ ^]^ bioceramics,^[^ ^15^, ^16^ ^]^ or decellularized ECM (dECM).^[^ ^17^ ^]^ BTE offers many innovative strategies to produce porous scaffolds such as those with tunable and programmable degradation behavior allowing them to alter their porous features during culture.^[^ ^4^, ^18^, ^19^ ^]^ While porosity is an inherent feature of trabecular bone, introducing controlled porosity into in vitro models of cortical bone is often also critical to facilitate osteoprogenitor cell spreading, migration, and 3D network formation.^[^ ^20^ ^]^ Beyond bulk porosity, surface topology plays a key role in directing cell behavior. For example, the curvature of microparticles has been shown to influence osteogenic differentiation, with smaller particles (165 µm diameter) promoting greater alkaline phosphatase (ALP) activity and osteogenic gene expression compared to larger particles (250 µm) or flat substrates.^[^ ^21^ ^]^ Additionally, both surface curvature—convex versus concave—and the spatial distribution of anchorage points have been reported to modulate cell behavior,^[^ ^22^, ^23^ ^]^ though these effects remain underexplored in the context of BoC platforms.

#### 2.1.3 Anisotropy and Structural Heterogeneity

Finally, bone is mechanically highly anisotropic, particularly in mature lamellar bone, where aligned collagen fibers impart direction‐dependent mechanical properties.^[^ ^12^, ^24^ ^]^ Although osteoblasts respond to the anisotropic nature of the 3D ECM‐mimicking environments,^[^ ^25^ ^]^ this anisotropic feature remains largely unexplored in BoCs. While one BoC investigation has shown that high interstitial fluid flow can modify the orientation of collagen fibers,^[^ ^26^ ^]^ BTE strategies provide a wider range of possibilities to achieve anisotropy in biomaterials including through mechanical shear, magnetic/electric fields, directed self‐assembly, and 3D‐printing.^[^ ^24^ ^]^ Bone's architectural features provide not only the framework for mineral deposition but also the spatial context for cellular interactions.

### 2.2 Cellular Composition

#### 2.2.1 Cellular Interplay in Native Bone

Bone development and function result from a highly complex cellular interplay. Bone formation begins with osteoblast‐driven formation of immature “woven” bone.^[^ ^11^ ^]^ This bone type is subsequently transformed to stronger, mature “lamellar” bone through remodeling enabled by the coordinated activities of bone‐forming osteoblasts, bone‐resorbing osteoclasts, and regulatory osteocytes.^[^ ^12^ ^]^

#### 2.2.2 Cell Culture in BoCs

While certain aspects of bone formation, resorption, or remodeling have been recapitulated in early‐stage BoCs, some applications may require mature bone, which remains unrealized.^[^ ^10^, ^30^, ^31^ ^]^ Moreover, despite the growing translational need to model dynamic aspects of bone biology, it remains a challenge to integrate the combination of key cell types involved in bone remodeling in BoCs. In particular, simultaneous co‐culture of osteoblasts, osteoclasts, and osteocytes—a prerequisite for modeling mature bone remodeling—remains largely unrealized. An osteopenic BoC model co‐cultured pre‐differentiated osteoblasts, osteoclasts and osteocytes, but it is unclear how stable the co‐culture was because they did not confirm the continuous presence of all three cell types during or after co‐culture.^[^ ^32^ ^]^ Most existing systems remain limited to monocultures, typically of osteoblasts, while osteocytes are often represented by murine cell lines such as MLO‐Y4^[^ ^33^, ^34^, ^35^ ^]^ or IDG‐SW3,^[^ ^36^ ^]^ rather than primary human cells. While on‐chip differentiation of osteocytes from human mesenchymal stromal cells (hMSCs) has been reported in an isolated instance, this approach requires prolonged culture periods (i.e., 19 months) which is technically challenging.^[^ ^37^ ^]^

#### 2.2.3 Cell Culture in BTE

BTE has yielded several cellular models that highlight principles directly translatable to BoCs. A self‐organizing osteoblast–osteocyte co‐culture has generated woven bone organoids,^[^ ^11^ ^]^ underscoring the importance of cell migration and fluid‐flow–derived shear stress in bone formation. Adapting this to BoCs would require controlled perfusion systems and could be extended by incorporating osteoclast precursors to initiate remodeling.

A triple co‐culture of osteoblasts, osteoclasts, and osteocytes demonstrated how paracrine signaling between these populations can be maintained while still allowing separate analysis.^[^ ^38^ ^]^ While pre‐differentiation of osteoclasts was necessary in static culture, BoCs could circumvent this limitation by establishing dynamic gradients of media supplements, enabling simultaneous support of distinct cell populations. Related studies using patterned gradients to direct osteogenic differentiation^[^ ^39^ ^]^ suggest similar strategies could be used to regulate bone remodeling on‐chip.

Long‐term differentiation of osteoblasts into osteocytes within fibrin hydrogels between posts highlights how defined physical constraints can yield aligned collagen fibers and osteocyte‐like networks resembling lamellar bone.^[^ ^40^ ^]^ Such approaches emphasize the role of scaffold geometry and mechanical tension as design elements for BoCs aiming to capture mature tissue organization.

Together, these BTE models show that self‐organization, multicellular signaling, and scaffold‐guided architecture are critical for recapitulating bone physiology. Integrating these lessons into BoCs offers a path toward platforms capable of coordinated remodeling and matrix adaptation.

### 2.3 Chemical Composition

#### 2.3.1 ECM Composition of Native Bone ECM

**Figure 2:**
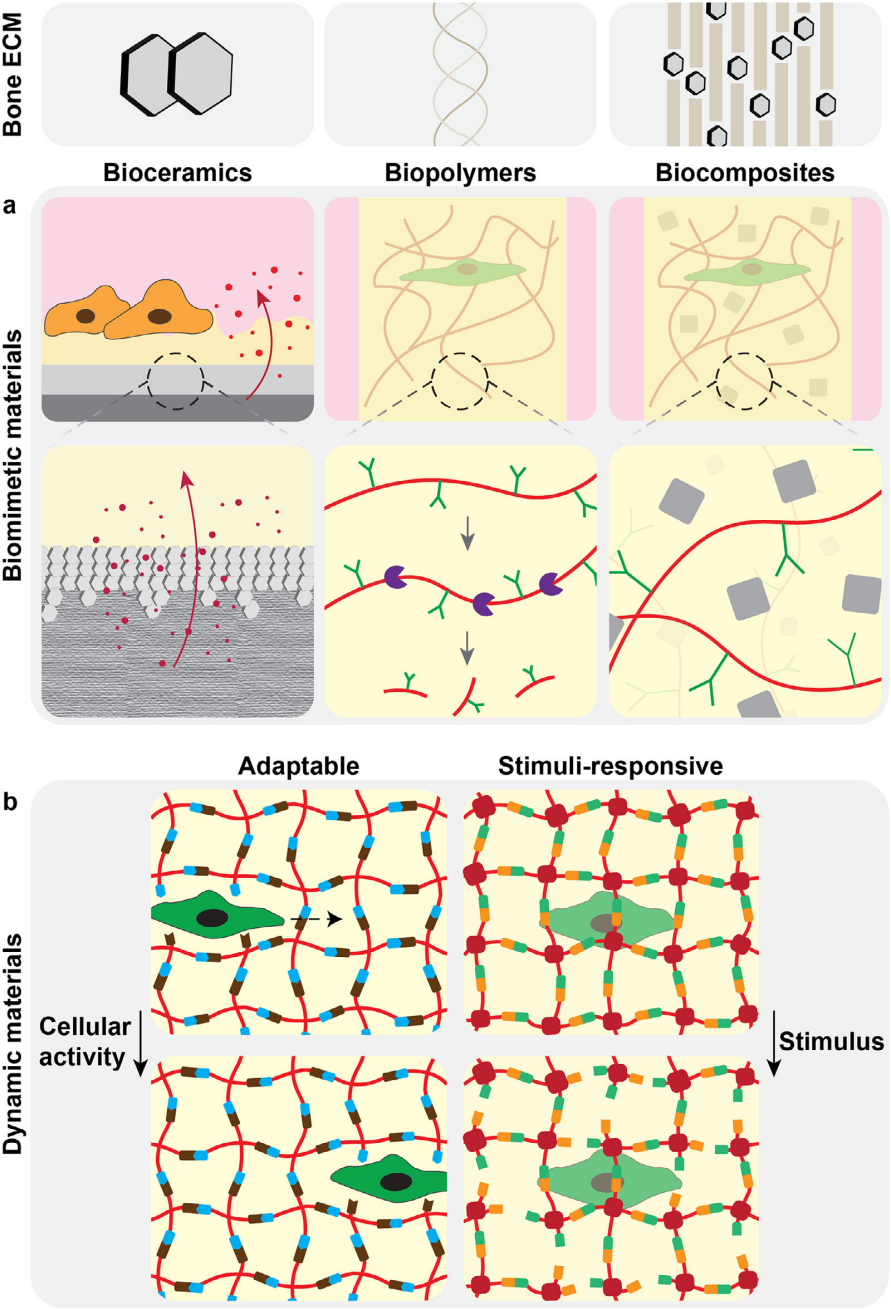
Overview of biomaterials for BoCs. Various biomaterials have been developed for BTE which can be employed as ECM mimics in BoCs recapitulating compositional features of native bone. a) Three key categories are biopolymers, bioceramics and their combinations in the form of biocomposites. While biopolymers can be designed to be biodegradable based on different mechanisms, bioceramics can allow for bone‐like mineralization and release of bioactive ions. b) Recent developments offer the emerging class of dynamic biomaterials, mainly in the form of hydrogels, that more closely recapitulate the dynamic features of bone formation and remodeling. Adaptable and self‐healing hydrogels allow cellular activities such as migration without requiring porosity or biodegradability.^[^ ^4^, ^57^ ^]^ Stimuli‐responsive biomaterials can degrade and/or release payload molecules upon user‐mediated or biology‐mediated stimuli such as light, temperature, pH or biochemical cues.^[^ ^18^, ^19^ ^]^ Such responses can be precisely programmed, e.g., by combinations of different crosslink types to function as logic gates.^[^ ^19^ ^]^

Bone ECM is an organic‐inorganic nanocomposite, which is mainly composed of collagen type I and hydroxyapatite (HA)^[^ ^4^ ^]^ (**Figure** **2**). This nanocomposite matrix forms in vivo through ALP‐mediated deposition of amorphous calcium phosphate (CaP) minerals within a collagenous matrix, followed by CaP crystallization into HA.^[^ ^11^ ^]^

#### 2.3.2 Inorganic ECM Mimics

To recapitulate the inorganic phase of bone, some BoC investigations have employed CaP‐based bioceramic coatings or scaffolds made of HA, or β‐tricalcium phosphate^[^ ^15^ ^]^ (Figure 2a). BTE research has established a wider range of bioceramics for bone regeneration, such as bioactive glasses which may be more effective for in situ formation of bone‐like HA and local release of bioactive ions.^[^ ^4^ ^]^

#### 2.3.3 Organic ECM Mimics

Other BoC investigations instead mimic the organic phase, utilizing a polymer matrix as the initial ECM mimic (Figure 2a). To this end, microfluidic channels have been i) coated with organic ECM mimics (e.g., laminin,^[^ ^7^ ^]^ poly‐D‐lysine,^[^ ^31^ ^]^ or collagen), or ii) filled with cells encapsulated in 3D polymeric matrices (e.g., collagen,^[^ ^8^, ^9^, ^26^, ^31^, ^41^, ^42^, ^43^ ^]^ gelatin methacryloyl,^[^ ^44^ ^]^ or fibrin^[^ ^40^, ^42^, ^45^, ^46^ ^]^). While natural collagen and its derivative gelatin are among the most cost‐effective and scalable cell‐binding polymers, BTE strategies offer numerous engineered synthetic and natural polymers exhibiting tailored functional properties.^[^ ^4^ ^]^ For example, various non‐covalent and covalent chemistries have been established for polymeric hydrogels, such as alginate rendered enzymatically degradable using enzymatically cleavable crosslinkers.^[^ ^47^ ^]^ In addition to degradability, cell‐biomaterial adhesion represents a critical design requirement for BoC biomaterials, particularly for anchorage‐dependent cells such as osteoblasts and osteocytes, where adhesion determines both cellular function and stability under perfusion. By contrast, suspension cells such as hematopoietic or various immune cell populations do not require matrix adhesion, but their inclusion in BoCs still necessitates careful consideration of the surrounding microenvironment. Engineering such microenvironments for BoCs can be achieved by employing natural polymers that inherently present adhesion motifs (e.g., collagen and laminin), and/or by functionalizing synthetic polymers with bioactive peptides (e.g., RGD).^[^ ^4^ ^]^

#### 2.3.4 Composite ECM Mimics

Given the nanocomposite nature of bone, a BoC ideally combines organic and inorganic phases, replicating this feature (Figure 2a). To this end, osteoblasts can be employed to produce bone ECM components, and collagen, gelatin and silk fibroin can facilitate HA mineralization within their matrix.^[^ ^4^, ^48^ ^]^ Alternatively, BoCs could employ organic‐inorganic composites as ECM mimics from the start, providing additional bioactive cues toward bone formation.^[^ ^4^ ^]^ Such composite systems can be fabricated using common techniques such as solvent‐casting and electrospinning,^[^ ^4^, ^49^ ^]^ through biomimetic strategies such as pre‐mineralization of polymeric constructs,^[^ ^4^, ^50^, ^51^ ^]^ or by incorporating bioceramic particles into a polymeric matrix.^[^ ^4^, ^52^ ^]^ More recently, particle‐based biomaterial design strategies have attracted growing interest, leading to the development of colloidal or granular systems.^[^ ^53^, ^54^, ^55^ ^]^ These materials offer unique advantages, including modularity (e.g., combining different particle types) and injectability, the latter being particularly relevant for integration into commonly used closed‐top BoC designs. Nonetheless, designers of BoCs must consider potential drawbacks such as the optical opacity of bioceramic phases and light scattering by particulate building blocks. These features may restrict characterization options, particularly for real‐time or live‐cell imaging.

#### 2.3.5 Matrix Remodeling and Degradation

The composition of native bone varies at different stages of formation or remodeling. This dynamic feature, however, has been largely overlooked in available BoCs but could be addressed using dynamic biomaterials. Adaptable and self‐healing hydrogels allow cellular activities such as migration without requiring porosity or biodegradability,^[^ ^56^, ^57^ ^]^ whereas stimuli‐responsive biomaterials can degrade and/or release payload molecules upon user‐mediated or biology‐mediated stimuli such as light, temperature, pH or biochemical cues (Figure 2b).^[^ ^18^ ^]^ A recent study showed that matrix degradation and architecturally‐controlled in situ pore formation using a matrix metalloproteinase (MMP)‐degradable hydrogel significantly enhance MSC network formation and osteogenesis.^[^ ^20^ ^]^ Similarly, MMP inhibition has been shown to significantly reduce human osteoblast motility, highlighting the importance of dynamic matrix remodeling for efficient cell migration in BoCs.^[^ ^45^ ^]^ Mineralization of the matrix can be enhanced with dynamic biomaterials functionalized with ALP, which mineralize the matrix even in the absence of active osteoblasts.^[^ ^58^ ^]^

The physicochemical properties of the ECM (mimics) in BoCs are also critical for accurately modeling various bone pathophysiologies. For instance, several studies have shown that ECM (mimic) characteristics strongly influence cancer cell invasion dynamics, underscoring the need for tissue‐specific and adaptable matrices in advanced BoCs to more accurately replicate organotropic invasion and enhance predictive capabilities for metastasis research.^[^ ^44^, ^45^, ^59^, ^60^ ^]^

#### 2.3.6 Growth Factors

Chemical strategies such as bio‐orthogonal click chemistry^[^ ^61^ ^]^ can be applied in BoC platforms for temporal matrix functionalization with bioactive molecules such as growth factors (GFs) or RNA.^[^ ^62^ ^]^ Such signaling molecules, including bone morphogenic protein (BMP), Vascular endothelial growth factor (VEGF), Wnt, insulin‐like growth factor (IGF) I and II, platelet derived growth factor (PDGF), and fibroblast growth factor (FGF) 1 and 2, represent a critical compositional feature of native bone which play fundamental roles in orchestrating bone formation and remodeling in vivo.^[^ ^12^ ^]^ However, culture media in BoCs commonly do not include biological GFs except for those present in fetal bovine serum. Several BoC investigations have employed receptor activator of nuclear factor kappa‐Β ligand (RANKL) or interferon gamma (IFN‐γ) for cultures of osteoclasts^[^ ^8^, ^15^, ^46^ ^]^ or osteocytes,^[^ ^36^ ^]^ respectively. While the miniature nature of BoCs decreases the required amount of GFs, it remains a challenge to mimic dynamic physiological GF concentrations in vitro by GF additions to culture media, given the short half‐life and high cost of various GFs.^[^ ^63^ ^]^ To address these problems, BTE strategies offer solutions to immobilize GFs into the ECM mimic,^[^ ^63^ ^]^ (over)express GFs using genetically modified cells,^[^ ^7^ ^]^ or utilize appropriate (co)culture conditions.^[^ ^64^ ^]^ In addition, GFs can be incorporated into biomaterials through physical adsorption, entrapment, or affinity binding. Whereas adsorption and entrapment are simple but typically limited to short‐term delivery, affinity interactions (e.g., with heparin) enable immobilization of GFs in their native form, while covalent conjugation–though effective–may compromise bioactivity.^[^ ^63^ ^]^

Stimuli‐responsive biomaterials^[^ ^18^ ^]^ offer more control over how GFs can be released, to better mimic the evolving bone microenvironment (Figure 2b). Enhanced mineralization can be achieved for example with biomaterials functionalized with MMP‐cleavable peptide linkers which release BMP once cells start producing MMP to remodel the ECM (mimic).^[^ ^65^ ^]^

BTE even offers stimuli‐responsive biomaterials with multiple synergistic modifications such as an alginate gel which enhances cell viability and proliferation with RGD peptides, followed by enhancing osteogenic differentiation through the release of bone forming peptide 1 (BFP‐1)‐laden mesoporous silica nanoparticles.^[^ ^66^ ^]^ Photo‐cleavable biomaterials may serve as a method for non‐invasive temporal and spatial control over where certain tissue types develop in the chip, for example by wavelength‐selective release of different GFs such as BMP‐2 and BMP‐7.^[^ ^67^ ^]^ Conversely, biomaterials with photo‐cleavable reactive groups can be used to chemically pattern an immobilized concentration gradient of for example VEGF on‐chip, providing detailed control over vascularization.^[^ ^68^ ^]^ The most advanced “autonomous” type of stimuli‐responsive biomaterials is defined by a feedback loop that modulates the material's response based on cues from the surroundings.^[^ ^18^ ^]^ Perhaps such materials could play a role in establishing the right balance of signaling molecules for bone formation and remodeling, e.g. by releasing varying levels of RANKL or osteoprotegerin (OPG) depending on the local RANKL:OPG ratio (see section 3.3).

### 2.4 Mechanical Environment

#### 2.4.1 Mechanical Properties of Native Bone

Beyond the chemical effects of the molecules in the ECM (mimic) on bone cells, the behavior of bone cells is also shaped by the physical forces they experience and the mechanical environment in which these physical forces interact with the ECM (mimic) and the cells.

**Figure 3:**
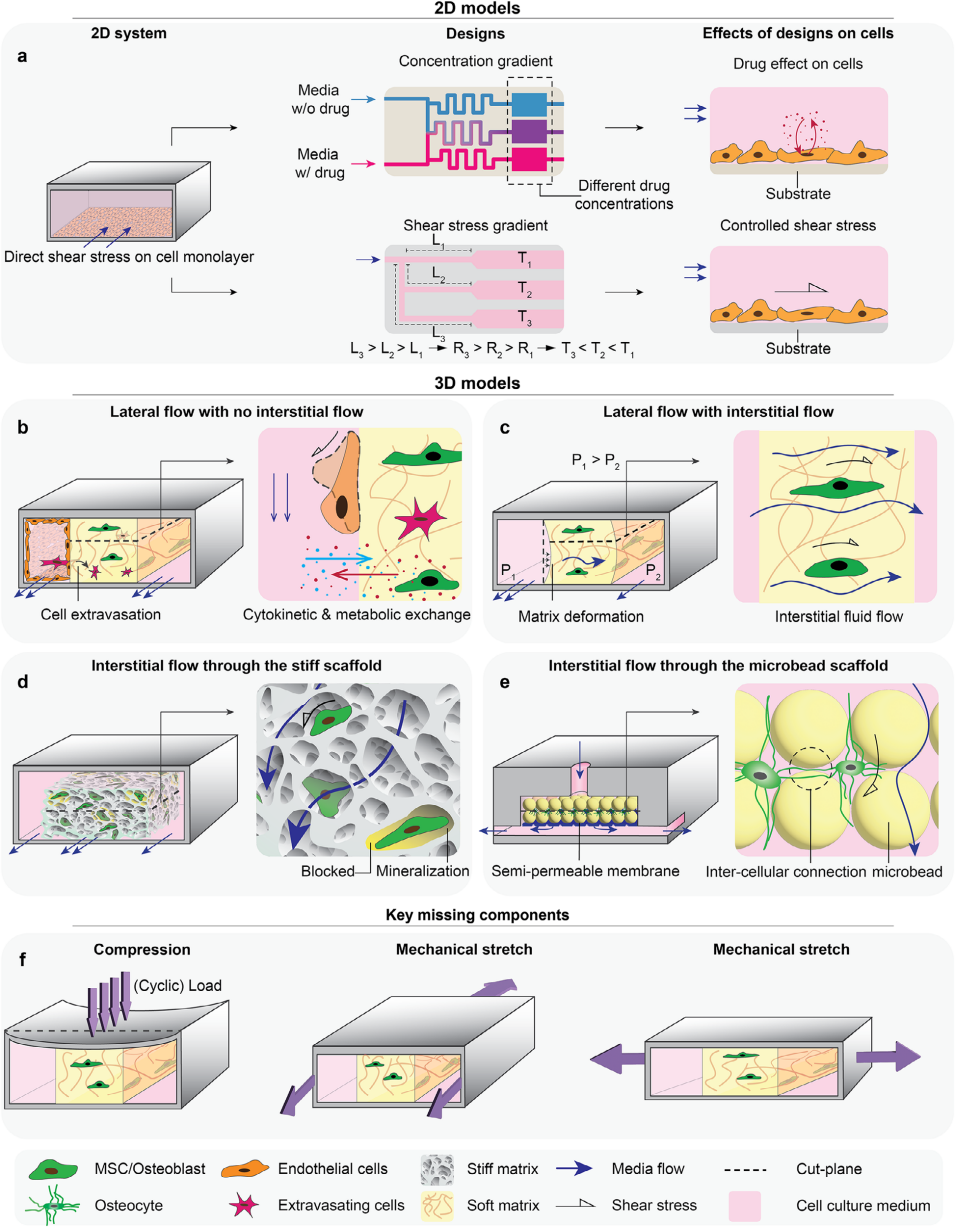
Overview of the state‐of‐the‐art of BoCs. Available BoCs can be divided into 2D and 3D models. a) 2D BoCs enable the study of effects of drugs on bone cell monolayers. A 2D gradient‐generating design^[^ ^9^ ^]^ allows for the creation of different drug concentrations in different channels by engineering channel bifurcations. This design can enable high‐throughput and low‐cost drug screening. 2D multi‐channel designs can also allow for screening the effects of different degrees of shear stress (τ_i_) on cells.^[^ ^8^ ^]^ This outcome can be achieved using different length (L_i_) channels with the same cross‐sectional area to control the flow rates of individual channels based on the channel resistance (R_i_) to the flow (i.e., channel geometry). b–e) 3D BoCs offer more realistic recapitulation of in vivo (patho)physiological conditions. Different types and combinations of flow are employed in these systems depending on specific requirements of the BoC. b) A BoC with lateral channels has been designed to study cancer cell extravasation into bone tissue through an endothelial layer.^[^ ^41^ ^]^ c) BoCs with lateral channels have also been employed to study the effects of interstitial flow on osteoblast migration.^[^ ^26^ ^]^ This configuration generates fluid flow through the matrix by creating a pressure (P_i_) gradient between two lateral channels. This design closely simulates in vivo interstitial flow, offering invaluable information on the effects of flow and hydrostatic pressure on the bone cells in a 3D BoC. d) 3D porous scaffolds are integrated in BoCs to study the synergistic effect of matrix stiffness and interstitial fluid flow on bone formation.^[^ ^13^, ^15^, ^17^ ^]^ However, mineralization in these systems may obstruct flow pathways, leading to imbalanced fluid flow and therefore a non‐uniform shear stress pattern. e) Alternative designs can be utilized to control factors such as inter‐cellular distance. For instance, a BoC design based on packed bioceramic microbeads has been employed to culture osteocytes at a defined inter‐cellular distance.^[^ ^16^ ^]^ f) One of the key missing components of existing BoCs is direct mechanical loading, which can be applied as cyclic compression of the top wall or as uniaxial stretching of the BoC to transfer desired strains to the embedded matrix and cells.

The mechanical features of bone can be divided into intrinsic (e.g., stiffness, viscoelasticity) and extrinsic (e.g., fluid flow, mechanical loading) features. Traditionally, BTE scaffolds for clinical application in heavily loaded skeletal sites are rigid (e.g., bioceramics and polymer‐matrix composites).^[^ ^4^ ^]^ However, current BoCs (**Figure** **3**) do not typically capture the load‐bearing nature of bone and consequently employ both rigid scaffolds^[^ ^14^, ^15^, ^69^ ^]^ and much softer matrices.^[^ ^26^, ^31^, ^45^ ^]^ The stiffness of the ECM (mimic), however, affects cell behavior.^[^ ^70^ ^]^ Common BTE strategies to tailor stiffness or viscoelasticity of ECM mimics tune the composition of organic‐inorganic composites^[^ ^4^ ^]^ or modulate crosslinking types in hydrogels.^[^ ^71^, ^72^ ^]^

#### 2.4.2 Matrix Viscoelasticity

While ECM (mimic) stiffness is one factor affecting cell behavior,^[^ ^70^ ^]^ cells indeed respond to the entire viscoelastic profile of the ECM (mimic).^[^ ^60^ ^]^ Nevertheless, current BoCs typically do not consider ECM (mimic) viscoelasticity. As the bone microenvironment exhibits different viscoelastic properties at different stages of formation or healing, such as fracture hematomas, replicating bone's varying viscoelasticity can enable superior recapitulation of the bone microenvironment.^[^ ^70^ ^]^ This aim can be accomplished through various dynamic hydrogel systems^[^ ^56^, ^60^, ^70^ ^]^ by tuning crosslink type and density as well as polymer chain length.^[^ ^60^ ^]^

#### 2.4.3 Interstitial Fluid Flow

Bone remodeling is regulated by several extrinsic biomechanical cues affecting bone cells.^[^ ^12^ ^]^ Convective fluid flow through vasculature and the lacunocanalicular network (LCN) – comprising osteocytes linked by intricate channels – supports bone metabolism and maintenance of cellular activities.^[^ ^59^ ^]^ Interstitial flow can also serve as a prominent regulator of bone remodeling^[^ ^73^ ^]^ in vitro primarily by inducing shear stress on cells. To mimic this perfusion, flow channels for culture media have been introduced in BoCs (Figure 3b). However, since side channels do not induce in vivo‐like shear stresses on cells,^[^ ^74^ ^]^ direct matrix perfusion has been explored in BoCs,^[^ ^20^, ^26^ ^]^ (Figure 3c–e). In such systems, the perfusion pattern and resulting local shear stresses can be affected by local matrix deposition and mineralization in long‐term cultures^[^ ^13^ ^]^ (Figure 3d). Consequently, long‐term cultures require specific attention to nutrient transport (e.g., through vascularization), and possibly dynamic mechanical stimulation to support bone cell functionality.

#### 2.4.4 Controlled Mechanical Loading

Direct mechanical loading is another key element in bone mechanotransduction, i.e., cellular sensing and response to mechanical signals. While externally applied forces induce strain in the bone, directly affecting cells, they also generate fluid flow that contributes an additional layer to the mechanotransduction cascade.^[^ ^69^ ^]^ Consequently, simulating in vivo‐like conditions requires accounting for the complex interplay between various experimental parameters, such as cyclic mechanical loading and interstitial fluid flow. In fact, studies have shown that the simultaneous modulation of mechanical forces and oxygen availability elicits cellular responses distinct from those observed under single‐variable control. For example, under physoxia (7% oxygen tension), intermittent mechanical loading enhances osteoblast proliferation and metabolic activity—both critical for ECM deposition. Yet, paradoxically, the same loading conditions under physoxic environments can also suppress mineralization,^[^ ^75^ ^]^ underscoring the importance of stage‐specific tuning of biomechanical and biochemical cues in different stages of model development.

Simplified bone models have illustrated the potential of mechanical loading alone to drive specific differentiation pathways. For example, continuous moderate mechanical tension, induced by cell‐mediated contraction between microposts has been shown to support osteoblast‐to‐osteocyte differentiation from primary human osteoblasts in osteogenic media.^[^ ^40^ ^]^ While such reductionist systems offer mechanistic insight into isolated stimuli, the integration of these cues within dynamic, multi‐input BoC platforms may enable more physiologically relevant control over bone cell behavior and tissue maturation.

#### 2.4.5 Electromechanical Stimulation and Piezoelectricity

Mechanical loading in native bone generates electrical charges due to its intrinsic piezoelectricity, which can modulate cellular activities directly or influence protein adsorption onto the biomaterial surface.^[^ ^76^ ^]^ Nevertheless, current BoCs have not yet actively incorporated electrical stimulation, whether by external devices or the use of piezoelectric materials. In BTE, however, this aspect has been explored in both in vitro and in vivo settings by altering the piezoelectric response of biomaterials such as collagen, hyaluronic acid, poly(vinylidene fluoride) and barium titanate through tuning of chemistry and porosity, as well as the directionality and amplitude of mechanical stimulation.^[^ ^76^ ^]^ Integrating such strategies into BoCs may provide an additional layer of physiologically relevant stimulation to more closely emulate the electromechanical environment of native bone.

## 3 Tissue‐Level Complexity of Native Bone

Together, the architectural, cellular, compositional, and mechanical features discussed above define the fundamental building blocks and processes of bone tissue. However, these features do not operate in isolation; in vivo, they are tightly coordinated through higher‐order multicellular and tissue‐level systems that regulate bone formation, remodeling, and adaptation to external stimuli. These dynamic systems include the basic multicellular unit (BMU), which orchestrates bone turnover; the vascular and neural networks, which provide metabolic support and regulatory cues; and the immune–bone interface, which integrates skeletal and immunological functions. Understanding and replicating these interconnected systems is essential for moving beyond reductionist models toward BoCs that capture the emergent behaviors of bone as an organ.

### 3.1 Bone Remodeling and the Basic Multicellular Unit (BMU)

#### 3.1.1 BMU Organization in Native Bone

Bone remodeling hinges on the activity of BMUs (Figure 1a, Cellular content).^[^ ^27^ ^]^ These transient anatomical structures consist of osteoclasts, osteoblasts, osteocytes, and supporting cells^[^ ^77^ ^]^ at various differentiation stages. Recent advances have illuminated the intricate spatial organization, biomolecular signaling, and mechanotransductive dynamics within BMUs, emphasizing their role as highly integrated systems—rather than mere isolated cellular assemblies—that orchestrate bone's dynamic and adaptable nature.^[^ ^27^, ^78^ ^]^ Despite their pivotal role in bone pathophysiology such as osteoporosis,^[^ ^79^ ^]^ our understanding of BMUs remains limited.^[^ ^77^ ^]^

#### 3.1.2 BMU Recapitulation in BoCs

The complexity of their structure and function, combined with ongoing debates surrounding their mechanistic operation, has hindered the development of in vitro models replicating distinct near‐physiological BMUs on or off chip.^[^ ^5^ ^]^ However, the capacity of BoCs for precise control of the architecture and physicochemical cues offers an unprecedented opportunity to study the BMU's role in bone remodeling and pathogenesis. For instance, by co‐culturing osteoblasts, osteoclasts, and osteocytes within a mineralized, architecturally defined matrix, BoCs may facilitate the in vitro recapitulation of BMU‐like structures. Since BMUs are spatially oriented processes requiring precise spatial and temporal control,^[^ ^27^ ^]^ BoCs offer a promising avenue for faithfully replicating these structures in vitro.

### 3.2 Neurovascular Networks

#### 3.2.1 Vascularization: Significance and Current Strategies

Vascularization and innervation play vital roles in various stages of bone formation and healing.^[^ ^80^ ^]^ Nevertheless, bone neurovascularization has been one of the least explored aspects in BoCs. In one parallel multichannel BoC, a lateral channel was utilized to mimic a blood vessel by incorporating an endothelial cell monolayer^[^ ^41^ ^]^ to study their interactions with bone cells. A recent study demonstrated that the angiogenic potential of cartilage during endochondral ossification (EO) is stage‐dependent: pre‐hypertrophic (immature) cartilage inhibits vascularization through anti‐angiogenic factors such as thrombospondin‐1 (THBS1), whereas hypertrophic (mature) cartilage promotes the formation of functional vasculature.^[^ ^81^ ^]^ These findings reflect the in vivo sequence of EO, where vascular invasion precedes the replacement of hypertrophic cartilage with bone. These results are further supported by evidence that osteogenic differentiation suppresses pro‐angiogenic signaling,^[^ ^82^ ^]^ highlighting the importance of temporally regulated vascular cues in BoC design.

#### 3.2.2 Vascularization: Proposed Strategies for BoCs

**Figure 4:**
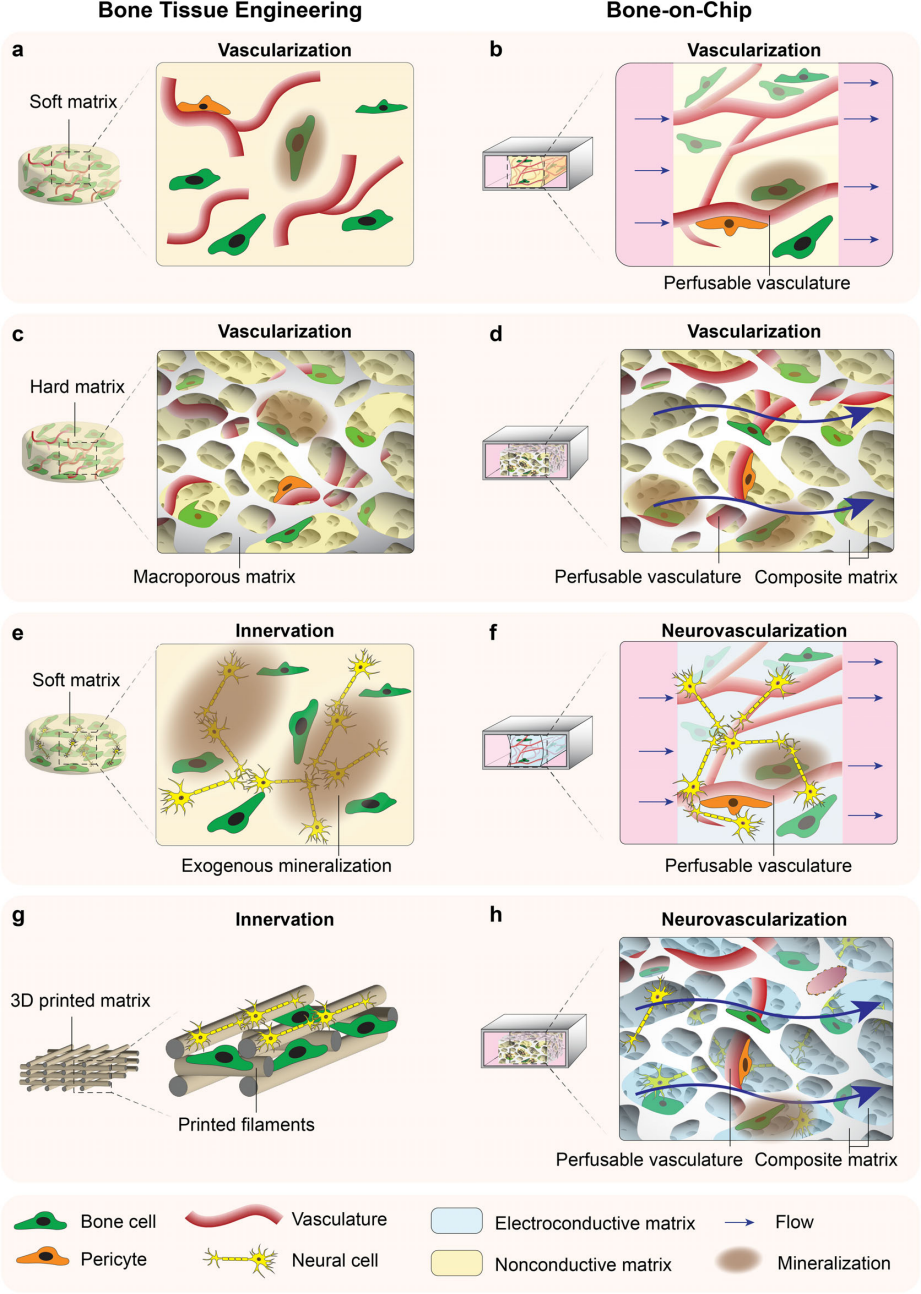
Schematic overview of key BTE neurovascularization approaches (left) and envisioned designs for BoC development (right). BTE approaches commonly generate vascularized bone tissue in a) a soft^[^ ^83^ ^]^ or c) a hard microporous matrix^[^ ^84^ ^]^ using MSC and HUVEC co‐cultures. The envisioned BoC counterparts translate this approach into flow‐mediated designs with perfusable vasculatures within b) soft matrices or d) composite matrices combining hard and soft regions. e) Innervation in BTE is typically pursued with soft matrices co‐cultured with MSCs and neuronal precursors, sometimes with exogenous mineralization to accelerate bone formation.^[^ ^84^ ^]^ The envisioned BoC design f) employs soft electroconductive matrices with tri‐culture of MSCs, neuronal precursors, and HUVECs under flow. Additional BTE strategies include g) 3D‐printed microporous composite matrices co‐cultured with MSCs, endothelial cells and neural precursors.^[^ ^85^, ^92^ ^]^ The corresponding envisioned BoC design h) integrates microporous hard matrices with embedded soft electroconductive matrices, and perfusable vasculature, supporting coordinated neurovascularization and near‐physiological bone development.

While several attempts have been made to fabricate vascularized bone models,^[^ ^30^, ^42^ ^]^ establishing an interconnected vascular network remains essential for achieving near‐physiological BoCs with a functional perfusion circuit to better mimic the biological processes of bone (patho)physiology. For example, co‐culturing human umbilical vein endothelial cells (HUVECs) with MSCs in soft hydrogels^[^ ^83^ ^]^ (**Figure** **4**a) or hard porous matrices^[^ ^84^ ^]^ (Figure 4c) is a common approach to induce vascularized bone formation in BTE.^[^ ^85^, ^86^ ^]^ However, perfusability of these systems are still suboptimal. In other OoC systems, perfusable vascular networks have been developed using interstitial flow or self‐assembling endothelial cells,^[^ ^78^, ^79^, ^80^ ^]^ offering potential solutions for vascular integration in BoC platforms.

Building on these observations, we propose two strategies tailored for BoCs. i) A promising approach is the use of MSC‐ and HUVEC‐laden matrices within a flow‐mediated system to induce endothelial self‐assembly, establishing perfusable vascular networks (Figure 4b). As the system matures, MSCs deposit ECM and flow‐aligned collagen fibrils that progressively mineralize, yielding a more stable, load‐bearing framework that supports long‐term perfusion. Vasculature stability can be enhanced by mural‐cell recruitment, with microvascular beds connected to macro‐inlets/outlets between patterned pillars. This strategy leverages soft matrices as dynamic, developmentally inspired frameworks in which vascularization and mineralization co‐evolve, resembling early stages of bone formation in vivo. ii) Combining soft and hard matrices in a flow‐mediated system offers another attractive strategy, particularly suited to a regenerative context where bone tissue forms around pre‐existing mineral phases (Figure 4d). In contrast to the developmental‐like strategy described above, this approach incorporates inorganic bone‐mimetic components from the outset of BoC culture. The soft matrix provides a permissive environment for vascular network formation, while the hard matrix delivers mechanical support and establishes a structural niche that enables studies of complex processes such as bone remodeling. For example, a porous HA scaffold could be infused with cell‐laden collagen that is crosslinked in situ, or alternatively, HA microspheres could be combined with cell‐laden collagen to form an injectable composite to introduce into the chip. Potential challenges include balancing vasculogenic and osteogenic media requirements, maintaining stable interfaces between mineral and hydrogel phases under flow, and ensuring uniform perfusion throughout the construct.

#### 3.2.3 Innervation: Significance and Current Strategies

Beyond vascularization, the sensory nervous system plays a vital role in regulating bone formation, remodeling, and repair^[^ ^87^ ^]^—yet it has received minimal attention in BoC design.

A recent study demonstrated that co‐culturing dorsal root ganglion neurons with MSCs on a parallel‐channel BoC separated by microgrooves activated canonical Wnt signaling in MSCs.^[^ ^88^ ^]^ Although conducted in 2D, this work highlights the potential of incorporating sensory neurons into BoC models to better capture neuroregulatory aspects of bone biology. Meanwhile, a limited number of BTE studies have focused on bone innervation.^[^ ^80^ ^]^ Particularly, matrix porosity, stiffness, and surface roughness in combination with different neuromodulatory biochemical factors and electroconductive matrices^[^ ^89^ ^]^ have been investigated.^[^ ^80^, ^90^ ^]^ Advanced fabrication techniques such as 3D printing has also been used where fabricated cell‐laden scaffolds (Figure 4g) were shown to sustain neuronal and vascular networks while enhancing bone regeneration in vivo.^[^ ^85^ ^]^

#### 3.2.4 Innervation: Proposed Strategies for BoCs

A major challenge in this field is the fact that engineering neuronal and bone cells in 3D requires different physicochemical biomaterial properties. This challenge might be overcome with two proposed designs: i) using electroconductive hydrogels combined with MSCs, neural precursors cells and HUVECs in a flow‐mediated system to support cellular differentiation (Figure 4f). The system can then be matured using osteogenic differentiation media, BMP‐2 supplementation, or controlled exogenous mineralization. Notably, post‐culture mineralization (Figure 4e) has been shown not to impair neuronal functionality.^[^ ^83^ ^]^ Potential challenges include ensuring that mineralization occurs in a spatially controlled manner and that neuronal networks remain stable during long‐term osteogenic induction; ii) designing BoCs using a scaffold composed of stiff and osteoconductive material with large pore size (average diameter 100–300 µm)^[^ ^91^ ^]^ filled with a soft electroconductive matrix containing neuromodulatory biochemical factors (Figure 4h). This architecture provides a load‐bearing mineralized framework while creating a permissive conductive niche for neuronal growth. Challenges include the potential need for exogenous electrical stimulation to enhance neuronal functionality, and balancing medium composition to sustain osteogenesis, vascularization, and neurogenesis simultaneously.

### 3.3 Osteoimmunology

#### 3.3.1 RANKL/RANK/OPG System in Native Bone

The immune system and skeletal system share extensive signaling networks, and their interactions play a crucial role in bone homeostasis, repair, and disease. Yet, osteoimmunology remains an underexplored dimension in BoC development. For a more comprehensive discussion of osteoimmunology, we refer readers to recent review articles.^[^ ^93^, ^94^ ^]^

**Figure 5:**
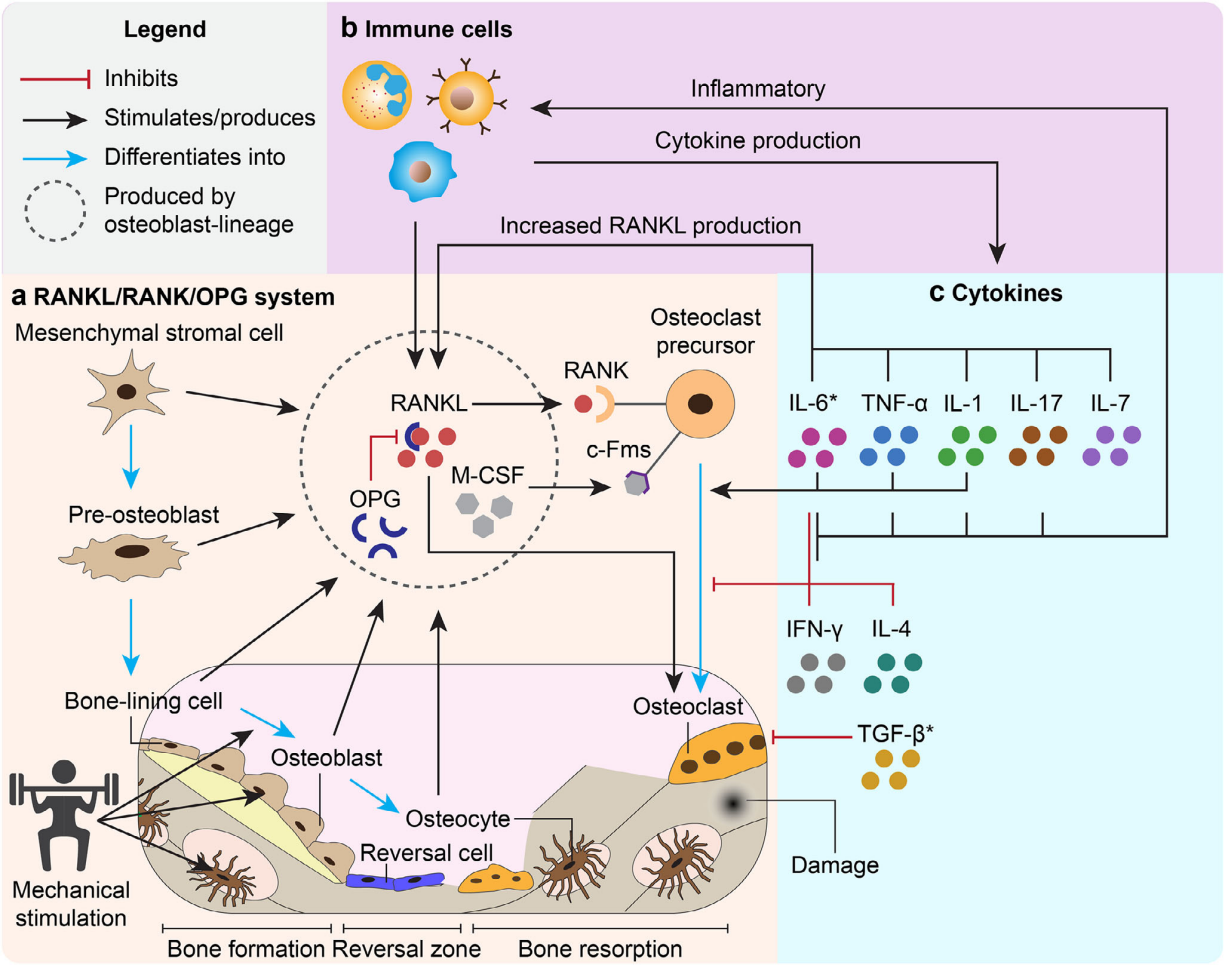
Overview of representative signaling pathways involved in osteoimmunology. a) Core regulation of bone remodeling through the RANKL/RANK/OPG system, M‐CSF and mechanical stimulation. b) Immune cells contribute to bone homeostasis by producing c) cytokines which modulate osteoclastogenesis either directly or by influencing RANKL, RANK, and OPG expression. *IL‐6 and TGF‐β are examples of cytokines with context‐dependent effects on bone remodeling (see section 3.3.3).

Bone remodeling is tightly regulated by the RANKL/RANK/OPG system (**Figure** **5**a). RANKL, produced by osteoblast‐lineage cells and immune cells (e.g., T cells), binds to RANK receptors on osteoclast precursors. RANKL–RANK signaling functions together with macrophage colony‐stimulating factor (M‐CSF) binding to c‐Fms (also known as colony‐stimulating factor 1 receptor (CSF1R)), driving osteoclastogenesis and bone resorption, and decreasing osteoclast apoptosis. OPG, a decoy receptor secreted by osteoblast‐lineage cells, inhibits RANKL, thereby suppressing osteoclastogenesis. How much resorption occurs is thus determined by the RANKL:OPG ratio, chiefly through their effect on osteoclasts and osteoclast precursors, and disruption of this ratio is a pivotal mechanism for bone loss in case of estrogen deficiency, inflammation, and cancer‐induced bone loss.^[^ ^93^ ^]^ Reversal cells, which bridge bone resorption and formation, respond to immune‐related osteoclastic coupling factors, thereby modulating osteoblast recruitment and differentiation during the remodeling process.^[^ ^95^ ^]^

#### 3.3.2 RANKL/RANK/OPG System in BoCs

The most common osteoclast precursors included in BoC models are monocytes^[^ ^10^, ^15^ ^]^ or RAW264.7 murine tumor macrophages.^[^ ^32^, ^96^, ^97^, ^98^ ^]^ RAW264.7 cells can differentiate into osteoclasts with RANKL stimulation alone as they secrete autocrine M‐CSF, saving the need for exogenous M‐CSF that other osteoclast precursors normally require.^[^ ^5^ ^]^

Nonetheless, reliance on exogenous RANKL is considered one of the key limitations of existing in vitro bone remodeling models, as it overrides the physiological RANKL:OPG balance that governs bone remodeling in vivo.^[^ ^5^ ^]^ Nevertheless, RANKL remains a standard component in most BoCs involving osteoclast precursors.^[^ ^8^, ^10^, ^15^, ^32^, ^96^, ^98^ ^]^ Efforts to stimulate osteoclastogenesis via endogenously produced RANKL have been limited by low and variable RANKL expression from osteoblast‐lineage cells, and/or by high levels of OPG, resulting in an inhibitory RANKL:OPG ratio.^[^ ^5^ ^]^ Overcoming this challenge may require cell sources that reliably express RANKL and the use of defined, serum‐free media.^[^ ^5^ ^]^

#### 3.3.3 Mechanical Cues and Immune‐Derived Cytokines

In addition to biochemical signals, mechanical cues sensed by osteocytes also modulate the RANKL:OPG balance, underscoring the importance of mechano‐immunological coupling in BoCs (Figure 5a). Mechanical stimulation promotes the differentiation of bone‐lining cells into osteoblasts, enhances matrix deposition, reduces apoptosis in osteoblasts and osteocytes, and modulates the secretion ratio of RANKL and OPG by these cells.^[^ ^93^ ^]^

In a BoC model for mechanotransduction studies, higher shear stress levels applied to osteocytes have been shown to reduce RANKL expression and subsequently suppress pre‐osteoclast differentiation in adjacent compartments.^[^ ^8^ ^]^ Beyond these cell‐intrinsic pathways, immune‐derived cytokines add an essential regulatory dimension to osteoclast behavior (Figure 5b,c). Cytokines such as tumor necrosis factor α (TNF‐α), transforming growth factor β (TGF‐β), interleukin (IL)‐1, IL‐6, and IL‐7 modulate osteoclastogenesis either directly or by influencing RANKL, RANK, and OPG expression.^[^ ^93^ ^]^ Among these cytokines, IL‐6 and TGF‐β exemplify the complex role that cytokines can play in the regulation of bone remodeling. IL‐6 exhibits a dual role in bone remodeling, acting as either a pro‐ or anti‐resorptive cytokine depending on the cellular composition, microenvironment, and stage of differentiation. In certain contexts, IL‐6 promotes osteoclastogenesis indirectly by stimulating RANKL expression, whereas under other conditions it can suppress osteoclast differentiation or enhance osteoblast activity.^[^ ^93^, ^99^ ^]^ Similarly, TGF‐β exerts context‐dependent effects on both osteoblasts and osteoclasts—stimulating proliferation or differentiation at early stages while inducing apoptosis or inhibitory signaling once bone resorption is complete.^[^ ^93^ ^]^

Collectively, these findings emphasize that mechanical and immune signaling are deeply intertwined in regulating bone remodeling, and that BoCs aiming to model such interactions must integrate both dynamic mechanical and immunological components. For a more detailed discussion of osteoimmunology and related signaling pathways, the reader is referred to recent comprehensive reviews.^[^ ^93^, ^94^, ^100^ ^]^

#### 3.3.4 Immune Components in BoCs

These cytokines are also implicated in pathological bone loss, making their inclusion in BoCs important for modeling disease‐relevant conditions. For instance, IL‐1β has been used in joint‐on‐chip (JoC) platforms to induce inflammatory microenvironments that mimic osteoarthritic joint microenvironments.^[^ ^101^, ^102^ ^]^ Cytokine supplementation enables the study of specific inflammatory cues without the added complexity of immune cell co‐culture. However, this approach simplifies the underlying biology. To the best of our knowledge, only one BoC model so far has incorporated monocytes to investigate monocyte extravasation across the synovium and accumulation of macrophages as a cause of osteoarthritis,^[^ ^45^ ^]^ and one BoC model has incorporated neutrophils to investigate the role of neutrophils in bone colonization by metastatic cancer cells.^[^ ^103^ ^]^

Current BoC models have thus included immune components through exogenous cytokine supplementation or limited co‐culture with immune cells. Future strategies for incorporating immune components in these systems include the use of conditioned media from immune cells to deliver paracrine cues,^[^ ^97^ ^]^ or coupling BoCs with bone marrow‐ or immune‐on‐chip platforms to enable more complex immunological interactions. The bone marrow serves as a critical microenvironment for immune system–bone crosstalk, but bone marrow and associated processes are outside of the scope of this perspective article. We refer readers to comprehensive reviews for in‐depth discussion on bone‐marrow‐on‐chip^[^ ^6^ ^]^ and immune‐system‐on‐chip, and the integration of immune components in OoCs.^[^ ^104^, ^105^, ^106^ ^]^

## 4 Bone Pathophysiology: From Critical‐Size Defects to Complex Disease Modeling

Developing bone‐disease‐on‐chip systems can enable new disease insights and innovative therapeutic solutions.^[^ ^41^, ^107^ ^]^ BTE strategies offer inspiration from various in vivo, ex vivo or in vitro models which can be adapted for BoCs modeling pathophysiological bone conditions.^[^ ^108^, ^109^ ^]^ Current BoC research efforts have primarily focused on modeling prevalent bone diseases such as osteoporosis,^[^ ^36^ ^]^ osteoarthritis,^[^ ^44^, ^45^, ^110^ ^]^ and bone metastasis.^[^ ^17^, ^41^, ^107^, ^111^, ^112^ ^]^ However, bone healing and a broader spectrum of skeletal disorders remains underexplored. For instance, BoCs could provide particularly useful platforms for studying developmental bone disorders,^[^ ^113^ ^]^ where access to patient‐derived tissue is limited and in vivo models are constrained. The features required in an in vitro model depend on the specific research question. In general, BoC models should remain as simple as possible —reducing cost, complexity, and confounding variables— while being as complex as necessary to capture the biological processes under study. Nonetheless, a BoC model that faithfully reproduces mature, healthy bone remodeling is likely to provide a strong foundation for many disease‐focused models. Key technical requirements for models of various levels of complexity for the most prevalent bone diseases are summarized in **Table** **1**.

**Table 1:** Overview of BoC models across healthy bone and major bone‐related diseases. The table provides a framework for BoC design based on the principle of “as simple as possible, but as complex as necessary” to address specific research questions. For each context, models of increasing complexity are outlined with basic requirements classified according to the classical BTE triad (cells, scaffold, bioactive cues), together with example use cases and literature references of existing BoC models. Non‐bone models, such as cartilage‐ or synovium‐only JoC systems, are not included.

(Patho)physiological context	Key (patho)physiological Features	Representative (patho)physiological focus	Basic requirements			Representative application	Ref.
			Cells	Scaffolds	Bioactive cues		
Healthy bone	Dynamic tissue undergoing formation, resorption, and remodeling; BMU (osteoblasts, osteocytes, osteoclasts); bone–immune crosstalk; vascularization and innervation	Bone formation	Osteoblasts or osteoprogenitors	3D matrix supporting osteogenesis (e.g., collagen with HA particles)	Osteogenic media; mechanical stimulation (e.g., fluid flow)	Study mechanisms of bone formation	[36]
		Bone resorption	Osteoclasts or their precursors	Resorbable, mineralized matrix (e.g., HA‐containing or pre‐mineralized via ALP/osteoblast activity)	Pro‐resorptive factors (e.g., RANKL, M‐CSF) to induce differentiation/activity	Study mechanisms of bone resorption	^*)^
		Bone remodeling	Co‐culture of osteoblasts, osteoclasts, and osteocytes; source of osteoclast precursors for longer‐term culture	3D resorbable, mineralized matrix allowing cell–cell communication (e.g., HA‐containing or pre‐mineralized via ALP/osteoblast activity)	Endogenous regulation of RANKL:OPG ratio; mechanical cues sensed by osteocytes (e.g., fluid flow)	Study mechanisms of bone remodeling	^*)^
Critical‐size defects	Defect too large for spontaneous healing; initiation of healing by fracture hematoma; involvement of hematopoietic, endothelial, and immune cells	Bone regeneration	^†)^	^†)^; 3D porous scaffold with a physical gap	^†)^	Test osteoinductive properties of biomaterials intended to fill the defect	^*)^
		Regeneration with hematoma mimic	^†)^; endothelial cells; immune cells	^†)^; biodegradable porous scaffold in the defect (e.g., fibrin/fibrinogen–thrombin)	^†)^; angiogenic factors (e.g., VEGF); inflammatory cytokines (e.g., TNF‐α, IL‐17)	Study regeneration, vascularization, and immune response to biomaterials	^*)^
Osteoporosis	Imbalance between bone resorption and formation; association with estrogen deficiency, aging, disuse, or long‐term glucocorticoid treatment; reduction in bone mineral density and microarchitectural deterioration	Formation	^†)^	^†)^	^†)^	Screen drugs that stimulate bone formation (anabolic agents)	[36]
		Resorption–formation imbalance	Bone remodeling model (§) (potentially simplified); adjusted cell ratios to mimic normal/osteopenic/osteoporotic conditions	^§)^	^§)^	Study remodeling imbalance; test anti‐resorptive and anabolic drugs; study effects of osteoporosis on metastasis	[32]
		Captures etiology	^§)^	^§)^	§; induce disease by mimicking causes: addition of glucocorticoids; removal of estrogen; mechanical unloading (with opposite controls)	Study mechanisms linking specific causes to osteoporosis; test drugs targeting specific etiologies	^*)^
Osteoarthritis	Progressive destruction of cartilage and subchondral bone; aberrant bone–cartilage crosstalk; contribution of mechanical stress, inflammation (e.g., IL‐1β, TNF‐α), and aging; immune‐cell infiltration of synovium sustaining inflammation; whole‐joint involvement (osteochondral unit, synovium, meniscus, ligaments, Hoffa's fat pad, immune cells)	Osteochondral unit	Chondrocytes; osteoblast‐lineage cells (optionally from the same MSC source)	Biphasic or compartmentalized construct (cartilage + bone regions)	Chondrogenic and osteogenic media; flow and/or compression; disease induction via pro‐inflammatory cytokines (e.g., IL‐1β) and/or hyperphysiological loading	Study bone–cartilage crosstalk; test anti‐inflammatory or chondroprotective drugs; investigate joint mechanobiology	[44, 116, 117]
		Osteochondral with synovial inflammation	Osteochondral unit plus synovial fibroblasts; monocytes/macrophages; ± endothelial cells	Multi‐compartment or modular microfluidic platform connecting tissue units; shared simulated synovial‐fluid circuit	Cues of osteochondral unit plus pathological stimuli (e.g., IL‐1β, hyperphysiological loading) applied to synovium	Study bone–cartilage–synovium crosstalk; assess synovium‐initiated inflammation and immune‐cell extravasation; test drugs targeting extravasation, crosstalk, or immune responses	^*)^
		Whole joint	Prior model plus compartments for meniscus, ligaments, and Hoffa's fat pad	Modular multi‐OoC platform interconnecting tissue units	Cues of osteochondral unit with synovial inflammation plus control over systemic and local stimuli; simulated synovial‐fluid and blood‐perfused circuits	Model whole‐joint pathophysiology; investigate auxiliary tissues; examine risk factors (e.g., obesity)	^*)^
Cancer (primary tumors and bone metastasis)	Primary bone cancers (e.g., osteosarcoma); frequent site of breast and prostate cancer metastasis; complex interactions with the tumor microenvironment; states of dormancy vs invasion; potential contribution of immune cells and vasculature	Primary cancer	^†)^, ^‡)^, or ^§)^; primary cancer cells (e.g., osteosarcoma)	^†)^, ^‡)^, or ^§)^	^†)^, ^‡)^, or ^§)^; interstitial flow; hypoxia	Study tumor invasiveness; study tumor–microenvironment crosstalk; identify therapeutic targets	[9]
		Metastasis / cancer invasion	^†)^, ^‡)^, or ^§)^; metastatic cancer cells (e.g., breast, prostate)	^†)^, ^‡)^, or ^§)^	^†)^, ^‡)^, or ^§)^; interstitial flow; hypoxia	Study cancer invasion, niche formation, and dormancy; evaluate drug resistance	[107, 111, 128]
		Vascularized and/or immunocompetent	^†)^, ^‡)^, or ^§)^; primary or metastatic cancer cells (e.g., osteosarcoma, breast, prostate); endothelial cells (vasculature) and immune cells	^†)^, ^‡)^, or ^§)^; Multichannel microfluidic chip supporting perfusion and cell migration/extravasation	^†)^, ^‡)^, or ^§)^; Perfusion profiles supporting extravasation; immune‐cell interaction cues	Model cancer extravasation into the bone niche; study roles of immune cells in metastasis and colonization	[17, 41, 103]

*) To the best of our knowledge, no BoC models currently exist in this category.

^†)^ See the basic requirements of a bone formation model.

^‡)^ See the basic requirements of a bone resorption model.

^§)^ See the basic requirements of a bone remodeling model.

### 4.1 Critical‐Size Defects

#### 4.1.1 Current Absence of BoC Models of Critical‐Size Defects

Critical‐size bone defects are defects that are too large to heal spontaneously during a patient's lifetime without surgical intervention.^[^ ^108^ ^]^ Despite the clinical significance of bone fracture healing, available BoCs do not yet incorporate this concept of critical‐size defects in their design.^[^ ^109^ ^]^

#### 4.1.2 Insight from Animal Models of Critical‐Size Defects

Future BoC designs, however, may adopt BTE strategies for investigation of bone regeneration in such defects.^[^ ^108^, ^109^ ^]^ These strategies typically involve generating bone defects or gaps, either in cultured bone segments in ex vivo models^[^ ^109^ ^]^ or directly within animal skulls and long bones in in vivo studies.^[^ ^108^ ^]^

Importantly, the definition of “critical size” varies significantly across species, anatomical locations, and experimental models,^[^ ^108^ ^]^ and cannot be directly applied to microengineered BoCs. As an initial design approach, however, BoC models may adopt scaling strategies derived from established in vivo models—for example, segmental defect lengths equivalent to 2–2.5 times the bone diameter have been commonly used to define critical‐size defects.^[^ ^108^ ^]^ Nevertheless, translating such geometric principles to BoC platforms will require systematic validation studies.

Moreover, since fracture hematoma formation plays a key role in initiating bone healing, BoC models of critical‐size defects may consider incorporating hematopoietic, endothelial, and immune cells to more accurately replicate the early regenerative microenvironment.^[^ ^109^ ^]^

### 4.2 Osteoporosis

#### 4.2.1 Etiology of Osteoporosis

Osteoporosis is caused by excessive bone resorption and impaired bone formation which is in turn typically caused by post‐menopausal estrogen deficiency, age‐related bone loss, disuse, or long‐term glucocorticoid treatment, resulting in reduced bone density and therefore an elevated risk of bone fractures.^[^ ^114^ ^]^ BoCs can enable study of cell‐cell communication between regulators of bone homeostasis such as osteocytes, osteoclasts and osteoblasts at physiological intercellular distances.

#### 4.2.2 Existing BoC Models of Osteoporosis

A high‐throughput BoC platform for evaluation of anti‐osteoporotic drugs has been developed, utilizing co‐cultures of osteoblasts and osteocytes.^[^ ^36^ ^]^ Nevertheless, this system lacks osteoclasts and does not capture the critical imbalance between bone formation and resorption, which are central to the pathogenesis of osteoporosis.

A more advanced tri‐culture model including osteocytes, osteoblasts, and osteoclasts has begun to address this imbalance by adjusting cell ratios to represent normal bone, osteopenia, and osteoporosis. Notably, however, the osteopenic bone model is the only true tri‐culture, while the osteoporotic and “normal” bone models entirely lack osteoblasts or osteoclasts, respectively. Therefore, this design oversimplifies bone physiology and does not account for upstream pathogenic drivers such as estrogen deficiency, mechanical unloading, or glucocorticoid exposure. Despite these limitations, such platforms provide valuable opportunities to test therapies that directly modulate bone‐resorbing or bone‐forming cells, and to examine how osteoporosis may exacerbate comorbidities. For example, the aforementioned tri‐culture model revealed that osteoporotic conditions increased vascular permeability and promoted breast cancer cell invasion, highlighting the broader relevance of BoCs for studying systemic effects of bone diseases.^[^ ^32^ ^]^

#### 4.2.3 Insights from Animal Models of Osteoporosis

In BTE animal models, osteoporosis is induced through methods such as: i) ovariectomy or orchidectomy to simulate postmenopausal osteoporosis due to estrogen deficiency, ii) mechanical unloading by immobilizing the animal or specific limb to simulate disuse osteoporosis, or iii) glucocorticoid treatment to generate glucocorticoid‐induced osteoporosis.^[^ ^114^ ^]^ Adopting the first two methods for BoCs would require including control systems that mimic physiological hormonal levels and/or mechanical loading. However, glucocorticoid treatment can, in principle, be broadly applied to BoC models that have achieved a state of bone remodeling.

### 4.3 Osteoarthritis (OA)

#### 4.3.1 Etiology of OA

OA is a whole‐joint inflammatory bone disease leading to gradual destruction of cartilage and underlying bone.^[^ ^115^ ^]^ A central element in this disease is the abnormal molecular crosstalk between articular cartilage and the underlying subchondral bone. This condition is driven by a combination of mechanical stress, genetic predisposition, aging, and metabolic factors, which trigger the release of degradative enzymes such as MMPs and inflammatory cytokines such as IL‐1β, IL‐6 and TNF‐α (Figure 4c).^[^ ^115^ ^]^ Among these cytokines, IL‐1β plays a particularly important role by initiating a cascade that amplifies the inflammatory response and induces the production of chemokines that recruit monocytes and macrophages to the joint.^[^ ^101^ ^]^ Subsequently, macrophage infiltration into the synovium sustains the inflammatory response.^[^ ^101^ ^]^ To recapitulate these processes in vitro, at minimum, a JoC must include osteochondral and synovial membrane units, while more comprehensive models may also incorporate the meniscus, ligaments, Hoffa's fat pad, and immune cells.^[^ ^110^ ^]^

#### 4.3.2 Existing BoC Models of OA

Comparisons of cartilage‐only models with osteochondral models show the importance of bone/cartilage cross‐talk. In healthy conditions, the bone component exerts a supportive effect on overlying cartilage, promoting chondrogenesis. In contrast, under inflammatory conditions, this relationship is reversed and the bone compartment exacerbates cartilage degradation.^[^ ^44^ ^]^ Notably, IL‐1β even incited a stronger catabolic response in the cartilage when applied to the bone than directly to the cartilage, highlighting the significance of inter‐tissue communication.^[^ ^116^ ^]^

On‐chip osteochondral models developed to date typically consist of separate bone and cartilage compartments. These systems have been realized using various strategies: a membrane separating the two compartments,^[^ ^117^ ^]^ adjacent hydrogel constructs placed in direct contact within parallel channels,^[^ ^101^ ^]^ or a single hydrogel‐based construct exposed to different culture media from opposite sides.^[^ ^44^, ^116^ ^]^ In addition, an osteochondral unit without compartmental separation has been developed has been developed to establish a concentration gradient of osteogenic versus chondrogenic supplements in cell culture medium and create a gradual bone–cartilage interface.^[^ ^118^ ^]^ Nonetheless, this system was aimed at tissue regeneration rather than osteoarthritis modeling.

Moving beyond osteochondral‐only constructs, a recent model incorporated osteoclasts and endothelial cells in addition to MSCs/osteoblasts. Careful control of culture conditions enabled bone and cartilage formation, sustained osteoclast activity, and endothelial remodeling of the ECM mimic forming a microvascular network.^[^ ^101^ ^]^ When stimulated with IL‐1β, the model exhibited OA‐like features and was applied to test IL‐1 receptor antagonist and Celecoxib, demonstrating distinct drug responses and highlighting its potential as a drug‐screening platform.^[^ ^101^ ^]^ Other efforts have focused on a cartilage‐only system,^[^ ^119^ ^]^ or cartilage–synovium combinations without bone.^[^ ^120^ ^]^ For example, a model with endothelialized synovium showed monocyte extravasation in response to chemokine cocktails and synovial fluid from OA patients, and enabled evaluation of chemokine receptor antagonists as inhibitors of this process.^[^ ^45^ ^]^

Despite these advances, current OA‐on‐chip platforms remain fragmented: Osteochondral and synovium‐based models exist, but none combine these critical tissue interactions, nor do they yet incorporate mature bone. Some systems have included immune cells or vasculature, but other key joint components such as meniscus, ligaments, and Hoffa's fat pad remain absent.^[^ ^110^ ^]^

A related JoC developed for rheumatoid arthritis included osteoblast/osteoclast bone culture with synoviocytes, but lacked cartilage, underscoring the challenges of integrating multiple tissues in a single platform.^[^ ^98^ ^]^

#### 4.3.3 Insights from Animal Models of OA

Modeling primary OA (i.e., naturally degenerative joint) in OA‐on‐a‐chip models is highly challenging considering this condition has never been induced in animal models, as it requires a long waiting period for natural development of OA. Similarly, while induced pluripotent stem cells (iPSCs) are attractive as a readily available source for generating diverse tissue types needed in OA‐on‐chip models, these cells yield tissues with immature phenotypes.^[^ ^44^ ^]^ Given that aging and genetics are major risk factors for OA, primary cells derived from older patients or cells expanded toward senescence (including iPSCs) may provide more relevant inputs for BoC platforms.^[^ ^121^ ^]^ By contrast, secondary OA has been induced in animal models by genetic modification, trauma, or injection of chemicals,^[^ ^115^ ^]^ offering a more straightforward path for recapitulation in BoCs. Indeed, on‐chip models that apply hyperphysiological pressure^[^ ^110^, ^120^, ^122^ ^]^ or introduce pro‐inflammatory cytokines^[^ ^44^, ^45^, ^101^, ^116^, ^117^, ^120^ ^]^ are analogous to trauma‐ and chemically‐induced animal models, respectively. For a more comprehensive discussion of JoCs, we refer the reader to recent reviews.^[^ ^110^, ^121^, ^123^, ^124^ ^]^

### 4.4 Cancer

#### 4.4.1 Primary and Secondary Bone Malignancies

Bone can be host to primary cancers such as osteosarcoma, and is also one of the most common sites of cancer metastasis, mostly from breast and prostate cancers.^[^ ^125^ ^]^ Current bone cancer‐on‐a‐chip systems have focused on metastasis of breast^[^ ^17^, ^41^, ^107^, ^112^ ^]^ and prostate^[^ ^111^ ^]^ cancers.

#### 4.4.2 Existing BoC Models for Cancer Modeling

BoCs provide a powerful tool to model complex biological processes or isolate specific mechanisms from systemic effects.^[^ ^126^ ^]^ For example, 3D BoC cultures were used to complement in vivo findings on how acidosis in the tumor microenvironment influences MSCs to promote osteosarcoma invasiveness. The model revealed that acid‐exposed MSCs enhance osteosarcoma cell escape from tumor spheroids, and blocking IL‐6 significantly reduced migration, similar to the in vivo scenario. This example demonstrates how OoC technology can elucidate in vivo results, offering mechanistic insights and potential therapeutic targets.

A more complex premetastatic niche model revealed how tumor‐secreted factors modulate the bone microenvironment, driving dormancy or invasion via MSC and osteoclast interactions, respectively. While designed to study lung cancer metastasis to bone, this approach is highly adaptable to primary bone tumors, offering a pathway for future studies on tumor progression and therapeutic interventions by exploring sequential interactions between distinct niches.^[^ ^96^ ^]^ The value of BoCs in fundamental research is further highlighted by a study linking vascular damage to increased bone metastasis in osteoporotic conditions,^[^ ^32^ ^]^ demonstrating how BoCs can uncover mechanisms that are difficult to capture using traditional models.

#### 4.4.3 Immune components in BoC Cancer Models

As BoCs evolve to more closely mimic the tumor microenvironment, some models have begun to integrate immune components. One notable example is a multi‐stage platform developed to examine neutrophil migration in the context of breast cancer metastasis to bone.^[^ ^103^ ^]^ This model was established in three stages, by first making a bone‐mimicking environment from osteo‐differentiated MSCs and endothelial cells, then seeding human vascularized breast cancer metastatic seeds, and finally circulating neutrophils through the matured construct.^[^ ^103^ ^]^ Given the growing interest in cancer immunotherapy, we anticipate that BoC models incorporating immune cells will play an increasingly central role in uncovering tumor–immune interactions within the bone microenvironment.

#### 4.4.4 Insights from in Vitro and Animal Models of Bone Cancer

As BoC platforms continue to evolve in complexity and physiological relevance for cancer research, insights from BTE cancer models offer valuable strategies for advancing the recapitulation of bone cancer in BoCs. 3D in vitro models of bone cancer have been developed using both scaffold‐based and scaffold‐free strategies, typically using similar scaffolds or hydrogels as current BoCs. For instance, a scaffold‐based 3D osteosarcoma model has revealed that HA mineral content in 3D is critical for mimicking in vivo‐like osteosarcoma signaling and drug response in vitro.^[^ ^127^ ^]^ Scaffold‐free BTE in vitro cancer models include the use of tumor spheroids, which can be more easily incorporated into BoCs for 3D recapitulation of cancer microenvironments.^[^ ^125^ ^]^ In BTE animal models, primary bone cancer is typically induced by injection of bone cancer cells into mouse bone marrow or the blood stream.^[^ ^108^ ^]^ Secondary tumors are induced by injection of a primary tumor into the relevant tissue (e.g., breast fat pad), which is followed by investigation of metastasis into healthy bone, implanted bone fragments, or bone‐mimicking scaffolds.^[^ ^108^ ^]^ While the available metastasis BoCs are based on direct insertion or perfusion of cancer cells, future systems could mimic BTE in vivo models by employing serially connected multi‐chamber BoCs containing mature bone tissue and tissue with the primary tumor.

## 5 Design of BoCs: Opportunities and Key Considerations

**Figure 6:**
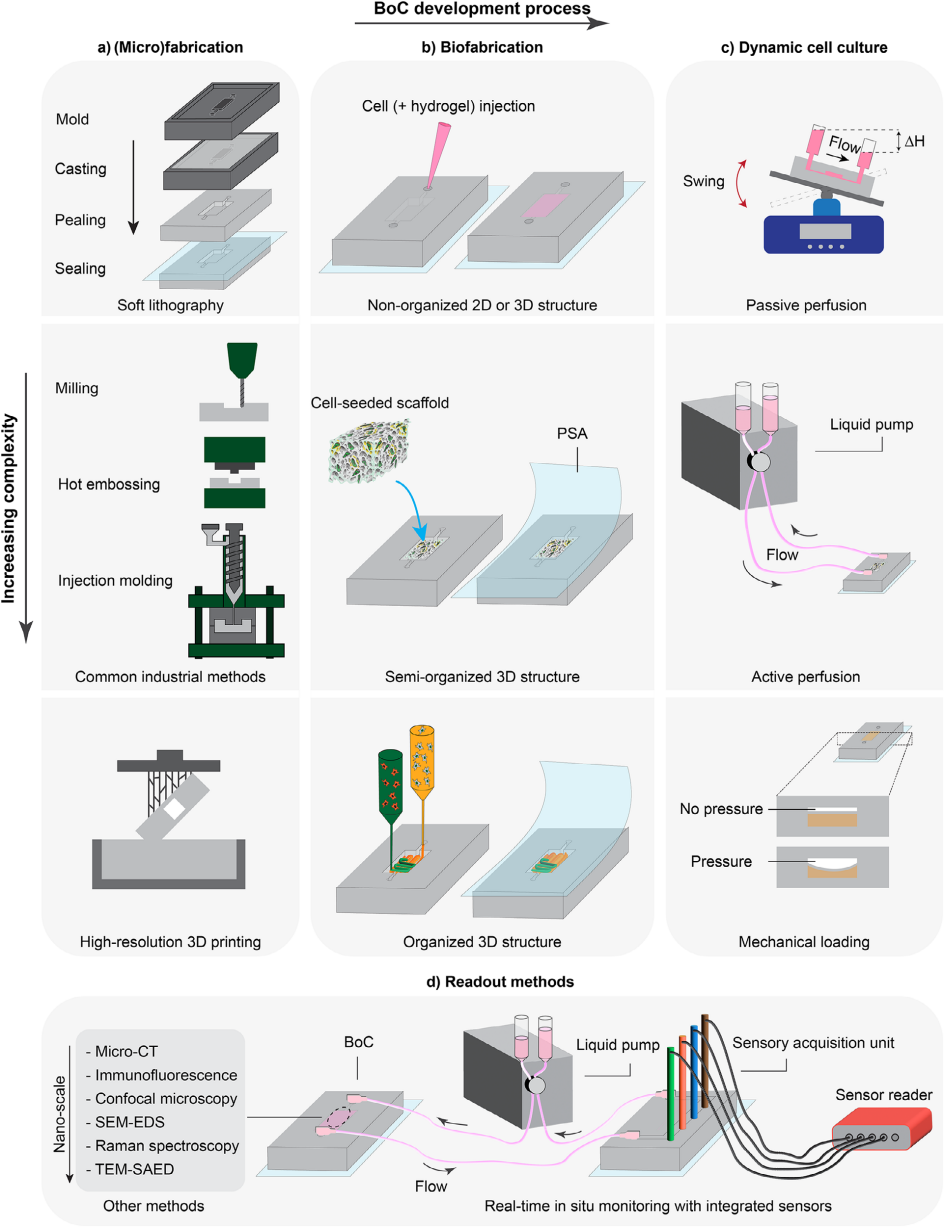
Overview of BoC development process. a) Microfabrication techniques for chip production with low‐ (i.e., poly(dimethylsiloxane) (PDMS) casting), medium‐ (i.e., computer numerical control (CNC) milling and 3D printing) and high‐throughput (i.e., injection molding and hot embossing). b) Biofabrication techniques for on‐chip generation of bone‐like cellular constructs. Top row) Injecting cells into the chip and allowing their attachment to the bottom of the culture chambers for 2D culture; injecting cell‐laden hydrogel precursor to the culture chamber and crosslinking afterward for a 3D culture. The chips can be made of PDMS and sealed using oxygen plasma for irreversible bonding, or of a hard material like poly(methyl methacrylate) (PMMA) and sealed with pressure sensitive adhesive (PSA) for reversible bonding. Middle row) Placing a cell‐seeded scaffold into a chip and sealing using PSA. This method directs cells to comply with the preformed architecture of the scaffold, which subsequently dictates the exact architecture of the cellular structure. Bottom row) Direct 3D bioprinting of cell‐laden hydrogels into chips can allow for higher complexity of the final tissue construct. It should be noted that some OoCs merge microfabrication and biofabrication steps to make devices from biomaterials such as hydrogels in one step. c) Methods for dynamic culture of BoCs. Top row) A rocker system relies on the height difference between the cell culture medium to start a flow which induces varying flow rates and thus varying shear stress levels during the swing cycles. Middle row) Controlled flow conditions can be achieved using precise pumping systems which can control the flow rates and shear stresses over the culture period. Bottom row) Application of mechanical stimulation to the cellular structure based on the membrane‐deflection method which uses a pneumatic source to pressurize the actuation chamber and deflect the thin elastic membrane to load the cellular structure. d) Readout methods for multiscale monitoring of BoCs. Schematic representation of real‐time in situ monitoring using a sensory acquisition unit housing multiple sensors (e.g., oxygen, lactate, pH, and electrical signals) which is directly integrated to a BoC through a pumping circuit enabling continuous monitoring of BoC functionality. In addition, complementary in situ/ex situ characterization methods such as micro‐computed tomography (micro‐CT), immunofluorescence microscopy, confocal microscopy, scanning electron microscopy with energy‐dispersive X‐ray spectroscopy (SEM‐EDS), Raman spectroscopy, and transmission electron microscopy with selected area electron diffraction (TEM‐SAED) provide architectural, chemical, and structural insights at different scales.

Novel methods for microchip fabrication and biomaterial design enhance our ability to dictate the structural and functional features of BoCs (**Figure** **6**). In the development of a BoC, principal factors that should be considered include i) culture architecture and scaffolding material, ii) cell types and number, iii) culture conditions, and iv) readout methods. When designing a BoC, it should be realized that increased model complexity is inevitably proportional to decreased ease of use, reproducibility, throughput, and ultimately widespread adoption. However, oversimplified models fail to accurately replicate the targeted biological process.^[^ ^129^ ^]^ Different purposes necessitate the design of application‐specific BoCs tailored to capture an essential level of complexity, rather than a universal one‐size‐fits‐all system. Consequently, the essential features of bone should be identified prior to inclusion in BoCs based on the particular application, striking a balance between physiological accuracy and practical considerations.

### 5.1 Accessible Chip Fabrication Methods

Fabrication of BoCs relies on the development of suitable chip architectures and materials. Unfortunately, optimizing chip design for development of BoCs often involves iterative testing. Common micro‐mold fabrication methods, such as photolithography, are time‐consuming and not widely accessible across different laboratories, hindering this process. Emerging 3D printing technologies, such as digital light processing (DLP), offer feature resolutions below 100 micrometers, providing a promising alternative that dramatically reduces device fabrication costs and time.^[^ ^130^ ^]^ Importantly, the expiration of key patents in 3D printing has made 3D printers increasingly affordable and accessible. In addition to fabrication techniques, the choice of chip material is also highly consequential in the device manufacturing process. The substitution of poly(dimethylsiloxane) (PDMS), the most common material for fabrication of OoCs, with alternative materials such as poly(methyl methacrylate) and poly(styrene) provides an opportunity to automate the production process of chips for BoCs. This automation can be implemented by substituting the manual molding process of PDMS with high‐precision methods such as computer numerical control (CNC) milling and hot embossing^[^ ^131^ ^]^ (Figure 6a). Moreover, elimination of the commonly employed permanent bonding step (i.e., plasma bonding) from chip fabrication allows for the use of open‐top or reversibly sealable BoC designs.

### 5.2 Engineering Tissue Structure and Interfaces

#### 5.2.1 Controlling Tissue Structure in BoCs

While the design of (micro)fluidic chips define the architecture of their chambers, the success of BoCs also depends on engineering the internal architecture and interfaces of the tissue models they host. By leveraging advanced biofabrication techniques employed in BTE, BoCs can be designed to incorporate some levels of bone‐like hierarchical complexity. One approach is 3D bioprinting, which provides superior control over architecture of the fabricated tissue model.^[^ ^4^ ^]^ Extrusion‐based bioprinting is the most widely used approach in BTE due to its low‐cost setup and ease of use.^[^ ^4^ ^]^ Nevertheless, extrusion‐based approaches are limited by their comparatively lower resolution. Alternative methods, such as stereolithography (SLA), DLP, and multiphoton lithography offer superior precision compared to extrusion. However, their practical use remains limited due to reliance on photopolymerizable bioinks with low cell‐loading capacity, cytotoxic photoinitiators, poor scalability to multicellular and multimaterial constructs, and complex, costly hardware requirements.^[^ ^132^, ^133^, ^134^ ^]^ In addition, these approaches typically involve substantial bioink consumption, which is particularly prohibitive when rare or expensive cell sources are used, restricting their utility for BoC applications in microscale.^[^ ^135^ ^]^

A practical compromise for the use of extrusion‐based bioprinting could be developing BoCs based on millimeter‐scale open‐top chambers, enabling direct bioprinting or integration of pre‐fabricated tissue‐like constructs.^[^ ^136^ ^]^ Such designs not only facilitate the incorporation of structurally diverse tissues within BoCs, but would also expand the range of biomaterials that can be incorporated in these systems (Figure 6b). These possibilities are particularly important given that current OoC systems are largely constrained to injectable soft matrices such as Matrigel™, which poses a significant barrier to the incorporation of more advanced or structurally complex biomaterials. Nevertheless, not all experimental objectives can be downscaled to micrometer dimensions (e.g., critical‐sized bone defect modeling), underscoring the need to balance architectural fidelity with practical constraints. As such, until high‐resolution bioprinting methods become faster, cost‐effective, widely available, and better adapted for multicellular biofabrication, a seamless integration of extrusion‐based bioprinting with BoCs, and OoCs in general, remains limited. In the near term, practical progress is more likely to be achieved through hybrid approaches by adapting chip designs to accommodate the capabilities of currently accessible bioprinting systems. For example, by employing open‐top BoC designs that can be sealed after the biofabrication step using various methods such as pressure‐sensitive adhesives (Figure 6b) or clamping in an external platform.^[^ ^137^ ^]^

#### 5.2.2 Recreating Tissue Interfaces in BoCs

For the recreation of tissue interfaces in BoCs, multichannel designs may be employed. For instance, pillar‐free designs with no separating pillars between adjacent chambers can be used for direct contact between different cell types in 3D, such as for studying the periosteum‐bone interface using two distinct 3D cell‐laden matrices. Alternatively, membranes can be implemented between channels to facilitate indirect cell interaction, paracrine signaling, and retention of secreted proteins for extended culture periods.^[^ ^107^ ^]^ For study of tissue‐biomaterial interfaces, chips should preferably allow for insertion of biomaterials after the on‐chip tissue development. This might be achieved using designs which allow for repeated access (e.g., open‐top) into the culture chamber for the introduction of non‐injectable biomaterials such as metallic implants, or parallel channel designs for the introduction of injectable biomaterials.

#### 5.2.3 Modeling Defects and Systemic Interactions in BoCs

Other designs could allow mimicking of bone defects. For example, a pillar‐free three‐parallel‐channel model might be used to simulate a bone defect by creating a bone‐like structures in the two lateral channels. The central channel could then be used to represent the bone defect, which could be filled with biomaterials or drugs to evaluate bone (re)generation in a controlled micro‐environment.

Finally, BoCs can be a valuable platform for studying the effects of cell‐secreted molecules on downstream tissues by designs allowing for the connection of different channels in a serial manner.^[^ ^96^ ^]^ This could be particularly useful to study the effects of cytokines in cancer metastasis to bone.

### 5.3 Controlling Culture Conditions

#### 5.3.1 Temporal Control of Biochemical Cues in BoCs

Controlling culture conditions such as chemical cues, perfusion and mechanical loading, while accounting for their temporal modulation, is crucial to develop near‐physiological bone tissue models.^[^ ^138^ ^]^ The introduction interval and concentration of biochemical factors can be controlled by their controlled inclusion in culture media, while spatial localization within BoCs can be dictated by employing semi‐permeable membranes.^[^ ^107^ ^]^

#### 5.3.2 Strategies for Incorporation of Mechanical Stimulation in BoCs

Mechanical stimulation can be incorporated in BoCs using methods established previously in OoCs,^[^ ^139^ ^]^ such as mechanical stretching or the membrane‐deflection method (Figures 3f and 6c). Previous studies suggest a discrepancy between in vitro and in vivo responses of bone cells to stresses resulting from similar levels of deformation.^[^ ^140^ ^]^ This discrepancy might be caused by fluid flow in the LCN which amplifies the stress levels on the bone cells in vivo, compared to the same strain levels applied directly to the cell cytoskeleton in vitro.^[^ ^138^ ^]^ Therefore, implementation of fluid shear stress in BoCs requires careful calculations with analytical or numerical methods^[^ ^17^ ^]^ to replicate near‐physiological mechanical stimulation. Importantly, it should be noted that identical flow rates can induce different shear stresses depending on the designed geometries^[^ ^141^ ^]^ (Figure 3a). In addition, due to the cyclic nature of fluid flow in the LCN (e.g., section 2.4), and reduced cellular response to continuous flow, cyclic fluid flow is preferable for near‐physiological BoCs.^[^ ^138^ ^]^ Moreover, matrix mineralization may hinder direct perfusion of the model over time in long‐term cultures (e.g., Figure 3d and Figure 4c,d,h).^[^ ^13^ ^]^ Therefore, such models are more likely to necessitate incorporation of vasculature in the BoC design (e.g., section 2.4 and Figure 4b,d,f,h).^[^ ^142^ ^]^

One technical setback of implementing fluid flow in BoCs involves the increasing complexity of the setup. Particularly, the increased amount of fluidic tubing may lead to elevated risk of bubble formation. This challenge can be overcome through unified fluidic platforms, which combine chips, pumps, connections, and bubble traps.^[^ ^143^ ^]^ For instance, this aspect has been addressed in BoCs by decreasing chip‐to‐medium‐reservoir distance, which was effective in minimizing such complications.^[^ ^10^ ^]^

#### 5.3.3 Dynamic Adaptation to Evolving Tissue Properties in BoCs

BoCs, akin to native bone tissue, exhibit temporal changes as cells and their ECM evolve during the culture period, through processes such as matrix remodeling and mineralization (e.g., Figure 4b,d,f,h). Therefore, external stimuli, such as mechanical loading and fluid flow, must be adjusted when maintaining consistent strain and stress levels throughout the culture period is desired. One possible approach involves measuring temporal changes of matrix mechanical properties using in situ force sensors and adjusting applied mechanical loads accordingly.^[^ ^144^ ^]^ As matrix mineralization progresses and the ECM stiffens during later stages of bone formation, commonly used mechanical stimulation methods—such as membrane deflection (e.g., Figures 3f and 6c)—may become inadequate, necessitating alternative strategies for effective mechanical loading. In parallel, ensuring uniform shear stress patterns throughout the system presents additional challenges. Newly formed bone typically occludes fluid channels (e.g., Figures 3d and 4c,d,h), resulting in increased local flow velocities at a constant flow rate, and thus higher local shear stresses.^[^ ^138^, ^141^ ^]^

#### 5.3.4 Oxygen Regulation in BoCs

In addition to mechanical and biochemical processes, oxygen regulation is yet another critical factor that, however, remains underexplored in BoCs. For instance, precise control of oxygen tension is pivotal for accurately modeling the bone–cartilage interface.^[^ ^145^, ^146^ ^]^ Chondrocytes typically reside under hypoxic conditions, while osteoblasts in vascularized bone require physoxia—making local oxygen control indispensable. The use of oxygen‐scavengers in micropatterned granular hydrogels has enabled the establishment of distinct oxygen levels for co‐cultured chondrocytes and osteoblasts. This approach successfully replicates sclerotic osteoblast‐induced changes in chondrocyte anabolic and catabolic activities, which uniform normoxic systems fail to capture.^[^ ^147^ ^]^ These findings underscore the broader importance of engineered oxygen gradients in BoCs—not only for osteochondral modeling, but also for recapitulating oxygen‐sensitive processes such as cellular metabolism, lineage‐specific differentiation, and hypoxia‐inducible factor (HIF)‐regulated signaling across bone‐associated tissue interfaces.^[^ ^148^ ^]^

### 5.4 Monitoring BoCs

#### 5.4.1 Imaging Considerations and Methods for BoCs

Capturing bone's multi‐scale complexity in BoCs requires readouts that span from the nanoscale to the macroscale.^[^ ^11^, ^149^ ^]^ The specific biological question determines the choice of readout method, which in turn influences the BoC design. Thanks to their miniature nature and common optical accessibility of their platforms, BoCs often offer the ability to evaluate the models using various microscopy techniques. However, specific considerations may be required for different applications. For example, high‐magnification imaging of a BoC requires the region of interest to fall within the objective's working distance. While software advancements—such as defocus particle tracking for wall shear stress analyses^[^ ^150^ ^]^—offer solutions for out‐of‐focus measurements, spatial constraints can still pose a challenge.

In addition to conventional microscopy, micro‐computed tomography (micro‐CT) enables non‐destructive 3D visualization of mineralized structures at the microscale.^[^ ^11^ ^]^ Confocal immunofluorescence microscopy provides spatially resolved detection of specific proteins or cell markers as well as high‐resolution 3D imaging of cells^[^ ^56^ ^]^ and ECM organization within BoCs. For surface morphology and elemental composition, scanning electron microscopy with energy‐dispersive X‐ray spectroscopy (SEM‐EDS)^[^ ^107^ ^]^ offers endpoint information at micro‐ to submicroscale resolution. This technique, however, is mainly limited to detecting inorganic elements in the ECM and does not typically yield detailed chemical fingerprints of the organic matrix. To obtain complementary molecular‐level information on both organic and inorganic components, vibrational spectroscopic approaches can be employed. More specifically, Raman spectroscopy^[^ ^11^ ^]^ enables label‐free, non‐destructive chemical fingerprinting of mineralization and biochemical composition in situ. Finally, transmission electron microscopy with selected area electron diffraction (TEM‐SAED)^[^ ^11^ ^]^ provides nanoscale to atomic‐level characterization of ultrastructure and crystallinity (Figure 6d).

#### 5.4.2 Biochemical and Molecular Analyses in BoCs

While the miniaturized nature of BoCs facilitates optical imaging, it also imposes limitations on sample volume, posing challenges for conventional biochemical and molecular analyses—including polymerase chain reaction (PCR)‐based methods, which typically require larger input volumes to ensure reliable detection. Therefore, BoC designs should carefully balance total sample volume and the cell–material–medium ratio to achieve sufficient cell density and signal concentration, as overly dilute conditions can compromise the accuracy and applicability of standard biochemical techniques. Moreover, such biochemical assays are often destructive and halt the experiment, providing end‐point information.

#### 5.4.3 In Situ Sensor Integration for Continuous Monitoring in BoCs

For continuous data acquisition, in situ measurements are preferable. To this end, although technologically challenging, many types of sensors have already been integrated into OoCs,^[^ ^151^ ^]^ and could be adopted by BoCs. For instance, integration of sensors to continuously monitor pH, oxygen tension, electrical and mechanical properties of a BoC system could significantly enhance BoC functionality (Figure 6d).

In addition to these direct integrations, sensing platforms developed within the broader lab‐on‐a‐chip field—which encompasses microfluidic devices designed for chemical and biological analyses that do not necessarily involve living cells—may offer complementary solutions for BoC monitoring (Figure 6d). For example, connecting a surface plasmon resonance microfluidic detector down‐stream of a BoC could allow for detection of low‐concentration biomarkers in real time.^[^ ^152^ ^]^ Finally, to facilitate widespread adoption, BoC designs should ideally enable plug‐and‐play sensor integration into standard platforms, thereby simplifying implementation and promoting reproducibility. A more comprehensive overview of biosensing opportunities for BoCs can be found in a recent review article.^[^ ^153^ ^]^

## 6 Qualification of BoCs: Application‐Driven Assessment for Translational Readiness

### 6.1 Validation and Qualification

In the absence of universally accepted standards for BoCs, these systems cannot be “validated” against accepted criteria but instead need to be “**qualified**” based on their intended application.^[^ ^154^ ^]^ Such qualification refers to demonstrating that a given BoC model is fit for its specific purpose, rather than meeting generalized or platform‐independent criteria. To this end, established biomarkers for bone formation and resorption are valuable measures.

### 6.2 Biomarkers for BoC Qualification

Bone formation biomarkers include deposition of collagen type I and CaP (e.g. HA), and levels of ALP and osteocalcin.^[^ ^155^ ^]^ Biomarkers for bone resorption include degradation of collagen and CaP as well as presence of tartrate‐resistant acid phosphatase (TRAP) and collagen crosslinking amino acids.^[^ ^155^ ^]^ However, confirming successful manufacture of BoCs solely from biomarkers is questionable. Therefore, assessing overall bone structure and morphology should also be included in such qualification. The structure of immature woven bone could for instance indicate bone formation,^[^ ^11^ ^]^ while the pits left by osteoclasts indicate bone resorption, and layers of parallel collagen fibers indicate remodeled lamellar bone.^[^ ^12^ ^]^ To qualify whether different bone diseases such as cancer and OA have been successfully mimicked, disease‐specific biomarkers or changes to the bone physiology should be specifically assessed.

### 6.3 Translation of BoC Prototypes to Commercial Products

Qualification of functional BoC prototypes however represents only the first of many steps to translation into a commercial product. Recognizing the multi‐faceted challenges in this pathway, the European Organ‐on‐Chip Society has presented a general roadmap to progress beyond prototypes in OoC research.^[^ ^154^ ^]^ The first stage in this pathway involves the creation of a customizable platform for fit‐for‐purpose, modular BoCs. Key elements in this step include development of standardized technical and biological modules, enhancement of throughput capacity, automation, development of multi‐parametric assays, use of well‐characterized human cells, and adoption of open technology platforms. Subsequent steps include standardization of BoCs and finally, large‐scale production and widespread adoption by stakeholders,^[^ ^154^ ^]^ setting the stage for the transformative impact of BoCs on biomedical research and healthcare systems.

## 7 Conclusions and Outlook

BoCs can serve as a versatile platform technology for biomedical applications including bone tissue modeling, bone disease modeling,^[^ ^14^ ^]^ and drug discovery.^[^ ^36^ ^]^ Current BoCs are at the stage of recapitulating selected features of bone, such as bone formation or resorption, while simplified bone‐disease‐on‐chip systems are also beginning to be developed. However, mature mineralized bone is a desired goal for development of near‐physiological BoCs and bone‐disease‐on‐chip systems. Progress in this area would greatly benefit from an approach that is both retrospective and prospective, as advancement of BoC platforms could greatly benefit from leveraging established BTE strategies as well as adopting emerging innovations. As previously stated, models should balance practicality with complexity to fit the intended application.

In addition to replicating key physiological features, practical considerations—such as ease of use, scalability, and high‐throughput compatibility—are crucial for broader adoption of BoCs in both academic and industrial settings. Designs that support user‐friendly operation, automation, and modularity can significantly enhance usability in drug screening and personalized medicine applications. Furthermore, versatility in material selection and applicable biofabrication methods is critical for accelerating progress. Enabling open‐top or repeated‐access BoC designs to accommodate a wide range of currently inaccessible biomaterials, including non‐injectable types, not only enhances physiological relevance but also facilitates the incorporation of advanced biomaterials developed in BTE, reducing the need for extensive redesigns and allowing for tailored applications.

**Figure 7:**
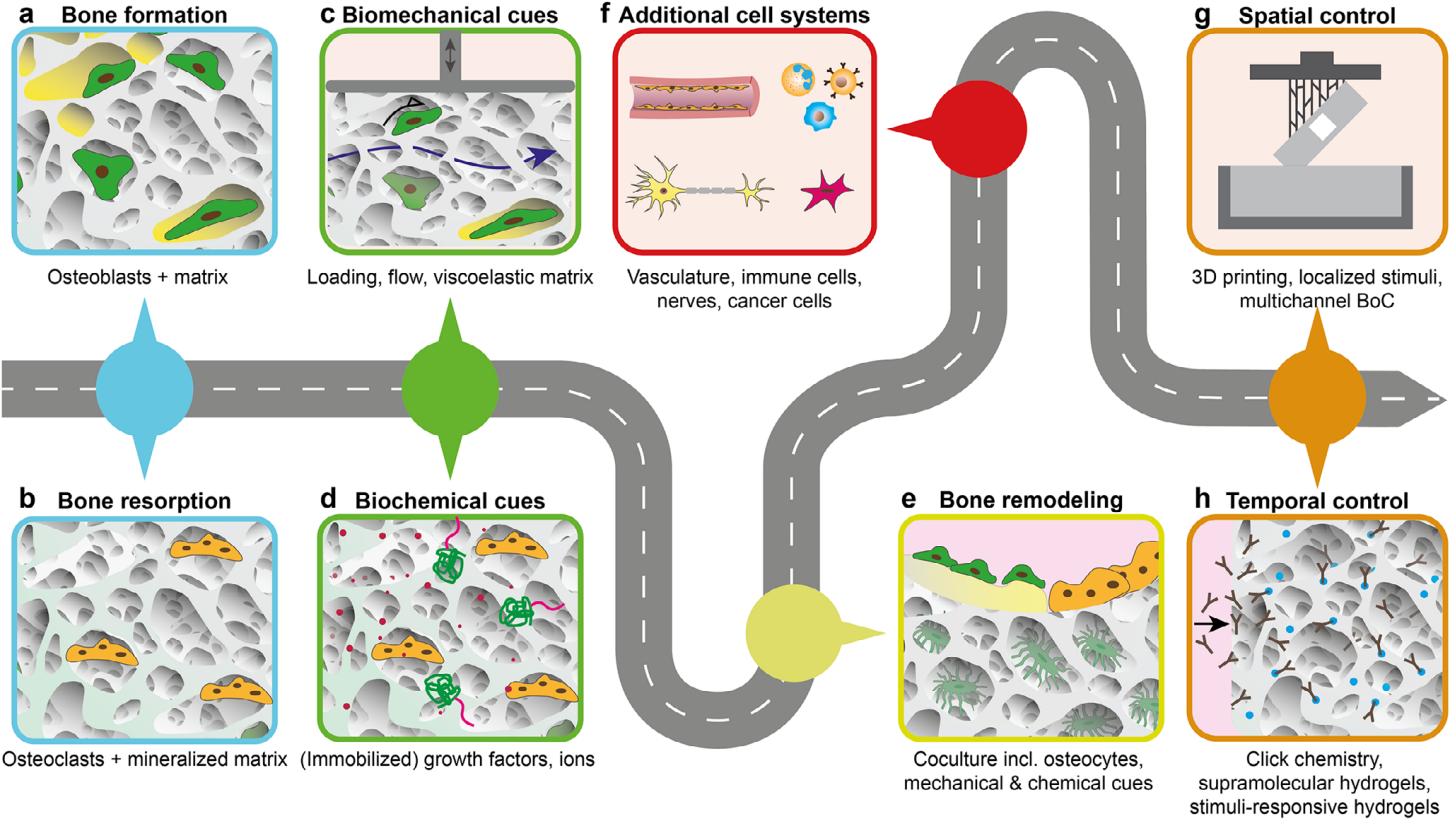
A conceptual roadmap for the development of next‐generation BoCs. Schematics illustrate: a) bone formation by (pre)osteoblasts and matrix deposition, b) bone resorption mediated by osteoclasts within a mineralized matrix, c) biomechanical cues such as direct loading, fluid flow, and viscoelastic matrix properties, d) biochemical cues including (immobilized) GFs and ions, e) bone remodeling through coculture of osteoblasts, osteoclasts, and osteocytes combined with mechanical and chemical cues, f) incorporation of additional cellular systems such as vascular, immune, neural, and cancer cells, g) spatial control via 3D (bio)printing, localized stimuli, or multichannel BoCs (e.g., for bone–marrow interactions), and h) temporal control through click chemistry, supramolecular assembly, and stimuli‐responsive hydrogels. While presented linearly for simplicity, this development process is likely to be concurrent across different stages. The illustrated sequence suggests a general prioritization of features, taking into account both their significance and implementation complexity for a range of applications.

We postulate that a path to near‐physiological BoCs starts with systematic recapitulation of the most important bone features, preferably beginning with the less complex targets (**Figure** **7**). Bone formation and resorption can therefore serve as the first targeted features (Figure 7a,b) as they require minimal cellular contentand a relatively short culture period, while providing a wealth of readouts. Bone formation requires osteoblasts and a matrix, whereas bone resorption requires osteoclasts and a mineralized matrix. This mineralized matrix can be provided as a pre‐mineralized scaffold or formed in situ by osteoblasts. While technically more challenging than static cultures, mimicking physiological bone formation requires a dynamic approach in which chip design and incorporated biomaterial facilitate matrix deposition and perfusion. This feature can be achieved by BoCs incorporating porous scaffolds, microbeads, or (3D printed) hydrogels (e.g., Figure 3b–e) in the culture chamber and a pump to facilitate medium perfusion. An open‐top single‐channel design for hard matrices or 3D bioprinted structures, or a closed single‐channel design for injectable biomaterials could facilitate this goal. Vascularization or innervation would typically require pores of 100–300 µm,^[^ ^91^ ^]^ but biodegradable scaffolds, such as those made of collagen, may only need smaller pores since the cells can degrade the matrix (e.g., section 3.2 and figure 4). In addition, various hydrogels can be designed to be biodegradable by inclusion of enzymatically cleavable crosslinks.

A more physiological but relatively complex next step in the development of BoCs involves capturing the biomechanical environment of bone (Figure 7c). This feature could be realized by implementing mechanical loading and/or perfusion as well as matrix viscoelasticity. A possible scenario for simultaneously implementing mechanical loading and perfusion entails integrating a deflectable membrane into a single‐channel chip design (section 2.4.2 and figures 6c and 3f). Similarly, the biochemical environment (Figure 7d) of native bone could be mimicked by including biochemical cues in the perfused media and/or matrix material, as well as by (over)producing them in situ using genetically modified cells.

Bone remodeling can be studied on‐chip once mature bone is formed (Figure 7e). To this end, BoCs should be created that incorporate co‐cultures of osteoblasts, osteocytes and osteoclasts with designs allowing their interaction under varying mechanical and biochemical conditions (e.g., section 5.3). These platforms can enable fundamental studies on the effects of drugs on bone formation, resorption and remodeling, possibly serving as a starting point for more complex bone disease models. Advanced bone disease models may require additional layers of complexity, such as the integration of vasculature and immunomodulatory features (Figure 7f). To facilitate clinical translation of such bone disease models into personalized medicine applications, relevant cell types can be differentiated from iPSCs derived from non‐invasively obtained patient samples. In addition, the use of iPSCs can enhance the reproducibility and standardization of BoCs, as these cells allow for long‐term expansion and provide a generally consistent cell source.

The subsequent step for enhancing the physiological relevance of BoCs is to control the architectural features of cellular content and ECM‐mimic (Figure 7g), although this approach often compromises the practicality of such BoC developments. These features could be realized by controlling the spatial distribution of cells, materials, and biochemical cues. 3D (bio)printing techniques offer a high degree of spatial control, but greatly limit biomaterial choice and typically require an open‐top chip design. Alternatively, a semi‐physiological spatial distribution of cells can be achieved directly using multichannel BoCs or indirectly through the spatial patterning of biochemical cues (e.g., section 2.3), allowing precise control over cell localization. The latter strategy might be achieved by exposure of a stimuli‐responsive hydrogel to localized stimuli, such as selective illumination of a photo‐responsive biomaterial (e.g., section 2.3.8); this approach, however, would require optically accessible chips.

Further steps might aim at recapitulating temporal changes in the native bone microenvironment within BoCs via on‐chip modulation of biomaterial properties (Figure 7h). To this end, conventional conjugation reactions between, e.g., succinimide and amines are applicable to a wide variety of biomaterials. However, these reactions are usually suboptimal due to incomplete yields and side reactions with biomolecules in/on cells which can disturb their normal function. Alternatively, bio‐orthogonal click chemistry allows for facile, non‐disruptive reactions on‐chip, but typically requires pre‐processing of the biomaterials to incorporate relevant reactive groups.^[^ ^61^ ^]^ Furthermore, self‐assembling supramolecular hydrogels might be tuned on‐chip through temporal introduction of co‐assembling components. Finally, dynamic biomaterials can be designed to change their properties in response to stimuli (e.g., section 2.3.7). Overall, BoCs with these dynamic features would greatly benefit from in situ monitoring strategies, for instance by integrating innovative sensors on‐chip (e.g., figure 6d).

In summary, the road to near‐physiological BoCs is paved with both challenges and opportunities. Harnessing insights from BTE offers promising solutions to overcome the existing challenges along this path, enabling innovative breakthroughs at both scientific and clinical levels.

## Conflict of Interest

The authors declare no conflict of interest.
